# Use of Health and Welfare Technology in Palliative Care: State-of-the-Art Review

**DOI:** 10.2196/79637

**Published:** 2026-03-12

**Authors:** Viktoria Zander, Maja Holm, Monir Mazaheri, Christine Gustafsson, Sara Landerdahl Stridsberg, Ragnhild Hedman

**Affiliations:** 1 School of Health, Care and Social Welfare Mälardalen University Eskilstuna Sweden; 2 Department of Nursing Science Sophiahemmet University College Stockholm, Stockholm Sweden; 3 Department of Health Care Sciences, Palliative Research Centre Marie Cederschiöld University Stockholm Sweden; 4 Department of Neurobiology Care Sciences and Society Nursing Division Karolinska Institutet Stockholm Sweden; 5 University Library Mälardalen University Västerås Sweden; 6 Stockholm Gerontology Research Center Stockholm Sweden

**Keywords:** digital technology, health and welfare technology, palliative care, state-of-the-art review, systematic review

## Abstract

**Background:**

As more individuals live longer with complex conditions, the need for effective palliative care (PC) grows. It has been stated that access to PC should be integrated early and delivered in a timely manner to patients with life-threatening illnesses. Health and welfare technologies (HWTs) offer tools to enhance care delivery, particularly in home and rural settings. Although there is a profound lack of evidence regarding the impact when used in PC, it is necessary to critically assess the current state of knowledge regarding impacts and consequences of technologies, ensuring that their integration considers broader implications for patients, caregivers, and health care systems in PC.

**Objective:**

This review explores health and welfare technology used in PC, aiming to inform practice and improve care quality.

**Methods:**

This state-of-the-art review included empirical studies describing the use of HWT in PC for adult patients. We used a thematic synthesis approach to compare studies and provide a synthesis of the key points.

**Results:**

Based on the inclusion criteria, 94 studies were included. PC is both a clinical specialty and an overall approach to care that focuses on improving quality of life and relieving suffering for patients and families facing serious illness, based on needs and not prognosis. HWT shows potential to increase access and continuity of care, for symptom management to support patients to remain at home and prevent frequent emergency visits. It can have the potential to build and remain relationships between patients, their families, and the health care team, as well as for interprofessional collaboration and support. However, there are challenges to overcome that might affect the quality of care when using technology.

**Conclusions:**

HWT shows potential as a complement to usual PC. Our findings point toward the importance of caution in choosing when to use HWT in PC, and for which patients.

## Introduction

As more individuals live longer with complex conditions, the need for effective palliative care (PC) grows. It has been stated that access to PC should be integrated early and delivered in a timely manner to patients with life-threatening illnesses. However, research has demonstrated that health care systems fail in this regard. Home-based PC is gaining attention, reflecting patient preferences to remain at home and potential cost savings from reduced hospitalizations. Health and welfare technologies (HWTs) offer tools to enhance care delivery, particularly in home and rural settings. However, questions about their feasibility and acceptability remain.

Today, an increasing number of individuals are living to older age with complex conditions and a great need for PC, including symptom relief and family support [1,2]. However, reports show that less than 15 % of patients requiring PC receive it in a timely and accurate manner [3]. PC is a holistic approach to the care of people with life-limiting conditions and near the end of life. It aims to acknowledge and attend to all aspects of the patients’ and family members’ needs when they are facing severe illness, including physiological, psychological, existential, and social issues. Symptom management, support of family members, and interprofessional teamwork are emphasized as crucial elements [3]. In recent decades, PC has evolved from primarily being offered to patients with incurable cancer, to currently being recommended to all patients with chronic and life-limiting illness [4]. Nonspecialized PC can be delivered by all care professionals, whereas specialized PC is provided in hospices, hospital PC units, and by specialized PC teams in home care [3]. Further, it has been highlighted that PC should be delivered early and integrated with specific illness treatment [5]. However, it has been demonstrated that access to PC varies and that patients with cancer still receive it more often than patients with other diagnoses [6,7].

Lately, there has been an increased focus on home-based care, especially since many patients wish to be cared for and die in their homes [8,9]. Home-based PC services offer many benefits, such as a sense of normalcy, choice, and comfort [10]. The prospect of dying at home is regarded as a more comfortable and dignified experience than dying in a hospital [10]. However, challenges exist in providing an optimal service. Unmet needs, uncoordinated care, and insufficient communication with health care professionals [11,12], as well as the demanding collaboration between specialists and home care professionals, make this challenging [13]. Moreover, home-based PC relies on the contribution of family caregivers [14], who often find themselves in a situation of managing multiple responsibilities and often forget their own needs to attend to those of the patient [10]. This informal care work often goes unnoticed and unaddressed [10,15]. From a societal point of view, home care has been associated with lower costs, as repeated hospital admissions are a major driver of expenditure in PC [16]. Although, the evidence for this is uncertain [17].

The COVID-19 pandemic has accelerated the demand for technologies in health care [18]. HWT comprises technology-based interventions that aim to maintain or promote health, well-being, quality of life, and/or increase efficiency in the operational delivery of welfare, social, and health care services, while improving working conditions for staff [19]. There are high expectations for HWT as solutions to challenges such as aging populations and limited resources [20-22]. HWT is expected to enhance the delivery and accessibility of PC, particularly in home settings [23] and in rural areas [24]. For instance, videoconferencing systems enable the remote delivery of multispecialty care, and artificial intelligence–driven wearable and nonwearable technologies facilitate remote assessments of patients’ conditions in their home environments, leading to more comprehensive clinical evaluations and empowering patients to monitor their own health [25]. Additionally, digital care plans can streamline home care delivery, potentially reducing avoidable hospitalizations [26].

Previous research has sought to gather evidence on the use of HWT in PC with a focus on telehealth (describing the provision of health care remotely by means of a variety of telecommunication tools and video consultations) [27-32], telemedicine (the use of remote technology and telecommunications) [33,34], eHealth (the use of information and communication technology for health care provision) [35], or with a focus on video consultations [36]. There is also specific focus on populations or settings, such as older adults [23], the professional perspective [30], or the patient perspective [29,35], home-based PC [23,32,37], or PC in rural areas [24,27,31,33]. However, there is a lack of literature reviews that have included the overall variety of HWT for all patients in PC and focused on impacts on patients as well as informal and formal caregivers.

Overall, HWT is frequently met with expectations, often based on biases, such as optimistic assumptions about what it could achieve and the belief that a technology will be as good as or better than a human, regardless of the task [38]. While this optimism emphasizes the potential for HWT, it often overlooks the complexities and challenges of implementing technology in sensitive areas such as PC [39]. There is also a profound lack of evidence of benefits and harms, as well as of impacts, when HWT is used in PC [40]. Other than telehealth, videoconferencing, or after-hours telephone support, there is little evidence for HWT used in PC [37]. Moreover, it is still unclear whether PC delivered remotely or with support of HWT is equivalent to more resource-intensive methods of in-person care [41]. Therefore, it is necessary to critically assess the current state of knowledge regarding the use of technologies for patients, caregivers, and health care systems in PC. This review explores HWT used in PC, aiming to inform practice and improve care quality.

This systematic review responds to the following research questions: (1) Which HWT is used in PC? (2) What impact does the use of HWT in PC have on patients and informal and formal caregivers? (3) What knowledge gaps and research needs are identified related to the use of HWT in PC? (The last research question is reported elsewhere).

## Methods

### Study Design

We conducted a state-of-the-art review. This type of review is appropriate to shape a comprehensive understanding of the current state of knowledge in a specific area [42]. The review followed PRISMA-ScR (Preferred Reporting Items for Systematic Reviews and Meta-Analyses extension for Scoping Reviews) [43]. The PRISMA-ScR checklist for this review is presented in Multimedia Appendix 1. A protocol was prospectively registered in the Open Science Framework on October 10, 2024 [44].

### Eligibility Criteria

Eligibility criteria were formulated in dialogue within the research group, based on the aim of the review. In line with Radbruch et al [4], PC was defined as holistic care of patients with severe illness, especially near the end of life, regardless of diagnosis and care place. Studies involving patients receiving curative treatment, or with the possibility of receiving curative treatment such as, kidney transplantation, were excluded.

Studies focusing on technology-based interventions for safety, activity, participation, and independence and/or increased efficiency in PC, and working conditions for professional caregivers were included. Further inclusion criteria were empirical studies (qualitative, quantitative, mixed methods, and case studies) describing the use of HWT in PC of adult patients (aged ≥18 years). The search was limited to studies published after 2012.

Studies that used technologies only for data collection were excluded, as were studies not reporting technology use in the intended setting. Study protocols, reviews, and studies describing the development of technologies without involvement of patients, informal caregivers, or health care professionals were also excluded, as were studies in languages other than English and studies that could not be obtained in full text.

### Information Sources

The following electronic databases were searched: PubMed, APA PsycINFO, Cochrane Library, CINAHL Plus, Scopus, and Web of Science Core Collection.

Searches were conducted on October 27, 2022. Additional update searches were completed on November 23, 2023.

### Search Strategy

Searches were conducted by an academic librarian (SLS). The search terms were organized to match the review’s goals, covering concepts such as PC, digital health and welfare technology, and home monitoring. Free-text words were searched in article titles and abstracts, along with database-specific subject headings, like MeSH (Medical Subject Headings) terms, when relevant. The search targeted articles published after 2012, excluding topics related to neonatal and pediatric care using the Boolean operator NOT to avoid content on children’s PC. Textbox 1 contains an example of search terms. The complete search strategies for all databases are available in Multimedia Appendix 2.

**Search strategy PubMed.**

**PubMed search strategy:**

- “Palliative Care”[MeSH Terms] OR “Palliative Medicine”[MeSH Terms] OR “Hospice and Palliative Care Nursing”[MeSH Terms] OR “Terminally ill”[MeSH Terms] OR “Terminal Care”[MeSH Major Topic] OR “Hospice Care”[MeSH Terms]
- “palliati*”[Title/Abstract] OR “hospice care”[Title/Abstract] OR “hospice nursing”[Title/Abstract] OR “terminal care”[Title/Abstract] OR “supportive care”[Title/Abstract] OR “terminal stage”[Title/Abstract] OR “terminal disease”[Title/Abstract] OR “terminally ill”[Title/Abstract] OR “end stage”[Title/Abstract] OR “end of life”[Title/Abstract] OR “hospice program*”[Title/Abstract] OR “advanced illness”[Title/Abstract]
- “eHealth”[Title/Abstract] OR “e-health”[Title/Abstract] OR “telemedicine”[Title/Abstract] OR “telehealth”[Title/Abstract] OR “mhealth”[Title/Abstract] OR “m-health”[Title/Abstract] OR “mobile health”[Title/Abstract] OR “e-homecare”[Title/Abstract] OR “digital health”[Title/Abstract]
- “Telemedicine”[MeSH Terms]
- “welfare technolog*”[Title/Abstract] OR “ambient assisted living*”[Title/Abstract] OR “ambient intelligence*”[Title/Abstract]
- “Ambient Intelligence”[MeSH Terms]
- “home monitoring”[Title/Abstract] OR “distance monitoring”[Title/Abstract] OR “distance care”[Title/Abstract] OR “distance nursing”[Title/Abstract] OR “distance medicine”[Title/Abstract] OR “environmental control*”[Title/Abstract] OR “remote sensing”[Title/Abstract] OR “Distance Counseling”[Title/Abstract] OR “Internet-Based Intervention”[Title/Abstract] OR “ambulatory monitoring”[Title/Abstract] OR “remote consultation”[Title/Abstract] OR “telecommunication*”[Title/Abstract] OR “E-Counseling”[Title/Abstract] OR “e therapy”[Title/Abstract] OR “distance spanning”[Title/Abstract] OR “health informatics”[Title/Abstract] OR “health information technology”[Title/Abstract] OR “medical information science”[Title/Abstract]
- “Remote Sensing Technology”[MeSH Terms] OR “Internet-Based Intervention”[MeSH Terms] OR “monitoring, ambulatory”[MeSH Terms] OR “Remote Consultation”[MeSH Terms] OR “Telecommunications”[MeSH Terms]

### Selection of Sources of Evidence

After the search, references were uploaded to Covidence, a web-based collaboration software for literature reviews [45]. Following the automatic removal of duplicates by Covidence and the manual removal of duplicates by the reviewers, a total of 3662 studies were examined for eligibility. Five of the authors (CG, VZ, MH, MM, and RH) conducted the abstract and full text screening. To start with, 40 abstracts were screened by 2 or 3 of the authors in various constellations, and their decisions to include or exclude were compared and discussed to ensure consistency. Subsequently, the abstract screening was equally divided among the authors. After the title and abstract screening, the remaining 601 articles were read in full and assessed for eligibility. A subset of 10 articles was initially read by all 5 authors separately and then compared. The remaining studies were divided among the authors and discussed when needed. All articles that matched the inclusion criteria were included in the review. In accordance with established practice for state-of-the-art reviews, no formal quality assessment of the included studies was undertaken [42].

**Figure 1:**
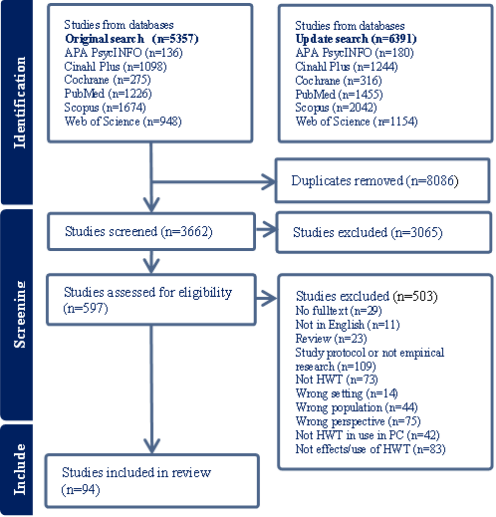
PRISMA (Preferred Reporting Items for Systematic Reviews and Meta-Analyses) flow diagram. HWT: health and welfare technology; PC: palliative care.

The screening process is described in a PRISMA (Preferred Reporting Items for Systematic Reviews and Meta-Analyses) flow diagram (Figure 1).

### Data Charting Process

After reviewing eligible studies, a data extraction template was generated to guide the data extraction process, including citation, study location, study aim and design, sample characteristics, intended target group, PC description, characteristic of technology, impacts of technology use in PC, knowledge gaps and research needs, and conclusions (knowledge gaps and research needs have been reported elsewhere).

### Synthesis of Results

Study characteristics were entered into an Excel (Microsoft Corp) spreadsheet. A thematic synthesis approach, led by VZ, supported by RH and MH, and discussed within the author group, was used to compare the studies and provide a synthesis of the key points. The process of the synthesis was guided by the first 2 steps described by Thomas and Harden [46]. These steps were (1) coding of the text—identification of themes across the included studies. Each study’s findings were identified and put into a metric. The findings were then coded line by line. Each sentence was read inductively to identify underpinning themes or concepts, which were labeled with a code; (2) developing descriptive themes—the descriptive codes were compared and organized into descriptive themes. Descriptive themes across the different categories of technology were compared and organized into one overall synthesis of the use of HWT in PC.

## Results

### Description of Included Studies

The selection process resulted in the inclusion of 94 articles describing the use of HWT in PC [47-140]. The included studies were published between 2012 and 2023. They were conducted in North America (Canada and United States; n=44), South America (Brazil and Chile; n=3), Australia (n=14), Africa (Kenya, sub-Saharan Africa, and Tanzania; n=4), Asia (China, India, Indonesia, and Taiwan; n=5), and Europe (Austria, Belgium, Finland, Georgia, Germany, Netherlands, Norway, Italy, Sweden, and United Kingdom; n=26). Some were cross-national. The review included studies using quantitative (n=50), qualitative (n=19), as well as mixed methods (n=25). The sample size of the studies ranged from 1 to 3178. The most common target group was patients, who were involved in 64 studies, followed by informal caregivers (involved in 31 studies) and health care professionals (involved in 21 studies). Several studies involved more than one target group. The most common diagnoses were cancer (n=38), followed by a few articles focusing on a variety of illnesses such as heart failure (n=2), chronic obstructive pulmonary disease (COPD, n=1), cirrhosis (n=1), dementia (n=1), and cognitive impairments in amyotrophic lateral sclerosis (n=1). Besides diagnoses, articles focused on older adults suffering from chronic illnesses (n=3), terminal illness and the end-of-life phase (n=12), and palliative care regardless of diagnosis (n=24). The characteristics of the studies included are presented in Multimedia Appendix 3.

### Health and Welfare Technology in Palliative Care

The HWT in the included studies were categorized based on function (Table 1). In some of the studies, HWT from more than 1 category were included, such as video consultations based on symptom monitoring.

**Table 1:** Categorization of health and welfare technology (HWT) in included studies.

Category of HWT	Examples of type of HWT
Symptom monitoring (n=25)	Systems to alert clinicians of changes in patient’s symptoms Symptom reporting systems and applications Systems for wireless data transfer Wearable monitoring devices
Telehealth consultations and conferences (n=51)	Videoconferencing Teleconsultations Telehealth-delivered care
Sharing of patient information (n=2)	Electronic care coordination systems Needs rounds via telehealth
Remote therapy and treatment interventions (n=7)	VR^a^ headset Therapeutic interventions in mobile apps Web-based therapeutic interventions Therapy delivered via email
Education and support (n=27)	Mobile apps and web-based education platforms for patients, caregivers, or health care personnel Teleconferencing technology to support and train health care personnel remotely Virtual webinar sessions Facebook support groups

^a^VR: virtual reality.

### The Use of Health and Welfare Technology in Palliative Care

#### Overview

The included studies described the use of HWT in PC on an individual level (including patients, informal caregivers, and health care professionals) and on an organizational level. The synthesis resulted in 7 themes, which are presented in the following subsections (Table 2 provides an overview).

**Table 2:** Descriptive themes and codes.

Descriptive themes	Codes
Symptom control and disease progression (n=26)	Impact on disease progression Impact on physical and mental symptoms Management of symptoms Disease-related stress
Quality of life and death (n=10)	Patients’ and caregivers’ quality of life Support in the end-of-life phase and death
Competence and palliative literacy (n=9)	Knowledge and understanding Self-efficacy Insight into norms and values
Palliative care provision (n=44)	Care satisfaction Medication use and compliance with therapies Prevention of excessive care Care coordination Accelerating advanced care planning Access to care
Multidimensional care relationships (n=18)	Partnership between patients and the health care team Companionship with and among significant others Interprofessional collaboration
Facilitating a comprehensive support system (n=33)	Professionals’ understanding Patient empowerment Support for the caring role
Organizational outcome (n=8)	Cost-effectiveness Time and travel Job satisfaction and distress

#### Symptom Control and Disease Progression

This theme focuses on the potential of HWT to support and prevent disease progression, physical and mental symptoms, and to relieve disease-related stress [47-72]. Types of HWT involved in symptom control and disease progression were technology for symptom monitoring [47,50,52-55,60,64,65,69,71], telehealth consultations and conferences [47,48,52,55-57,60,61,63,64,66-68,70], remote therapy and treatment interventions [58,59,62,72], and technology for education and support [49,51,56].

Among the included studies, the potential of HWT to support and prevent disease progression was mainly reported and discussed by 2 studies. Kazankov et al [47] described how a digital health solution with monitoring of heart rate, blood pressure, weight, body water, and cognitive function, for individuals with cirrhosis could facilitate timely intervention to prevent disease progression. Jiang et al [48] described how a model with a communication platform resulted in less functional decline. A total of 3 studies explored the effects of HWT on mortality, none of which could show any significant effects [49-51].

The impact of HWT on physical and mental symptoms was described by several studies. Positive impact on physical symptoms related to cancer illness and treatment was described by Tumeh et al [52], Mooney et al [53], Besse et al [54], and Cornetta et al [55]. Ngoma et al [50] reported no differences in overall symptoms when using remote symptom assessment and care coordination, although there were effects on symptom severity. Mark et al [56] reported a positive impact on dyspnea with an online teaching intervention for persons with COPD. Rafter [57] showed that an eHealth system allowed hospice professionals to deliver more effective care, with improvement in pressure ulcers for end-of-life patients. Impact on mental symptoms, such as anxiety and depression, was reported in 7 of the included studies. Positive effects were reported in cancer care studies using symptom monitoring [52], a virtual reality headset [58], a mindfulness intervention [59], telemedicine [60], videoconferencing [61], and web-based psychotherapy for informal caregivers [62]. On the other hand, one study using teleconsultations showed no differences in depression scores and increased anxiety scores [63]. Eight of the included studies showed the potential of HWT to contribute support in symptom management [64-71].

HWT was also used to relieve disease-related stress. For example, studies showed that an online support system in the care of patients with cancer has the potential to reduce symptom distress [49], mindfulness via video could reduce cancer-related stress [59], telehealth music therapy had positive affective effects [72], and teleconsultations resulted in lower distress symptom scores [68]. Maguire et al [69] showed that using a remote symptom monitoring system provided reassurance about symptom experience and the feeling of being listened to. However, Hoek et al [63], on the other hand, reported that adding weekly teleconsultations to usual PC led to a higher total distress score among home-dwelling patients with advanced cancer.

#### Quality of Life and Death

This theme describes patients’ and caregivers’ quality of life [52,56,60,69,73,74] and support in end-of-life phase and death [75-78]. It involves HWT for symptom monitoring [52,60,69], telehealth consultations and conferences [52,56,60,74,75,77,78], sharing of patient information [76], remote therapy and treatment interventions [73], and education and support [56,74].

Five of the included studies showed some effects on patients’ and informal caregivers’ quality of life with the use of HWT for symptom monitoring [52,60], training delivered over Skype for patients with COPD [56], a supportive care mobile app intervention [74], and a cognitive-behavioral therapy mobile app for anxiety [73]. Maguire et al [69] found that a system with daily symptom reports provided reassurance, but it did not lead to changes in quality of life.

Outcomes regarding support in the end-of-life phase and death were reported in 4 of the included studies. The potential to support dying at home or in another community setting was reported using an electronic PC coordination system [76], home care delivery with point-of-care technology and remotely located health care professionals [78], and a PC after-hours telephone number [75]. Johnston et al [77] reported the potential of Skype calls with dying persons and family members to bring closure and reconciliation, inclusion in the dying process, and healthy grieving.

#### Competence and Palliative Literacy

Competence and palliative literacy refer to knowledge and understanding, self-efficacy, and insight into norms and values [74,75,79-85]. This theme includes HWT for symptom monitoring [80,81], telehealth consultations and conferences [74,83], remote therapy and treatment interventions [85], and education and support [74,75,79,82-84].

Different HWT interventions have been shown to increase knowledge and understanding of health, diagnosis, symptoms, and concerns among patients [74,79], informal caregivers [80,81], and health care professionals [82,83]. An e-learning intervention for nurses to increase knowledge of PC and attitudes toward dying patients and death showed positive effects [82]. Teleconferencing technology to support and train hospice nurses remotely was perceived to improve knowledge as well as self-efficacy in caring of patients in PC [83]. The intervention had given them access to education that otherwise would have been difficult to obtain due to geography. Self-efficacy to manage their own condition was reported by patients with advanced cancer using a digital support app [75]. Digital information and education were also shown useful for addressing insights into one’s own thoughts and values related to advanced dementia among patients [84] and related to caring for a person with advanced cancer among informal caregivers, including accepting experiences of negative thoughts and feelings and being more aware of personal values [85].

#### Palliative Care Provision

PC provision includes subthemes such as care satisfaction [50,87], medication use and compliance with therapies [72,73,81,82,87,88], prevention of excessive care [48,51,64,65,68,69,76,86,89-98], care coordination [75,83,94,102-110], accelerating advanced care planning [51,84,87,93,94,97,99-101], and access to care [68,74,96,106,108,111-117]. This was mainly related to use of technology for telehealth consultations and conferences [48,64,68,74,75,83,84,87,88,91-96,98,100,102-104,106,108,109,111-116], but also technology for symptom monitoring [50,64,65,69,81,89,96,98,106,107,110,117], education and support [51,74,82-84,86,90,97,99,101,117], sharing of patient information [76,100], and remote therapy and treatment interventions [72,105].

The use of HWT was shown to be promising in the provision of care, including care satisfaction [50,87] and medication use and compliance with therapies [73,82,87,88]. Zeiser et al [72] reported that music therapy telehealth services increased compliance with other therapies. Other studies reported decreased polypharmacy and potentially decreased adverse drug events [88], but no effects on analgesic adherence [82] and no differences in anticipatory medication prescribing [87].

Some studies reported positive impacts on care coordination [102], care provision [83], increased efficiency [103-108], increased care resource use [75], and positive outcomes related to quality of care [94,106]. For example, Groothuizen et al [109] reported that virtual team meetings resulted in increased flexibility, reduced travel time, and easier real-time access to patient information for health care professionals. Health care professionals in the study by Oelschlägel et al [110], on the other hand, found that organizational challenges made it difficult to obtain and share the information necessary to provide seamless and optimal service to patients.

Prevention of excessive care is a core purpose of technology in PC and relates to the use of hospice care, emergency care, and hospitalization. Among the included studies, no effects were shown on hospice enrollment or length of stay [51], nor on time until entry into hospice [98]. Emergency department visits and admission could be avoided or decreased in several studies [65,69,76,86,89,90,92]. The HWT used in these studies varied. Technology to support patients (symptom monitoring and means to communicate needs to health care professionals) showed positive effects [65,69,89,92], as did technology used to support health care professionals in their work [76,86,90]. Manz et al [51] showed that opt-out text messages to prompt serious illness conversations decreased end-of-life systemic therapy relative to controls, but there was no effect on hospice enrollment or length of stay, inpatient death, or end-of-life intensive care unit use. Nor did a teleconsultation service with a triage system change the number of emergency visits [91]. HWT to support patients in communication with health care professionals [48,91,94,96] showed potential to decrease the need for hospital referrals. Unnecessary hospital transfers could also be prevented by improved collaboration among physicians using telemedicine [95]. Moreover, an advance care planning video program intervention increased documented “Do Not Hospitalize” orders among nursing home residents with advanced illness, but did not significantly reduce hospitalizations [97].

Technology was also used to accelerate advance care planning, with promising results in 7 studies [51,84,94,97,99-101], but no results in 2 studies [87,93].

Other positive effects reported were increased access to care [69,96,106,108,111-114] and increased continuity of care [113,115,116]. HWT was shown to enable health care professionals to respond quickly to patients’ care needs [96,106], allowing them to reach more patients [68,111], especially during epidemics or when out of town. For patients and informal caregivers, the technology increased the sense of security related to the ability to contact the clinic when needed [96,117].

#### Multidimensional Care Relationships

Multidimensional care relationships concern the ability to use HWT to form partnership between patients and health care teams, companionship with significant others, and interprofessional collaboration [65,69,77,96,104,110,114,116,118-127]. HWT used in the studies related to this theme included technology for symptom monitoring [65,69,96,110,119,120], telehealth consultations and conferences [77,96,104,110,114,116,118,119,122,127], and education and support [121,123-126].

Whether remote care can affect the partnership between patients and health care teams varies between studies and within studies. Rosa et al [118] described positive as well as negative impacts on the quality of relationships with patients, families, and between health care professionals. For health care professionals, increased availability to patients and their caregivers was positive, but there was a perceived loss of nonverbal cues, which made communication more difficult. Some health care professionals also experienced difficulties being supportive remotely during difficult times. It also put a strain on relationships among coworkers. Other studies reported no negative effects on relationships [114], enhanced connectivity with the care team [70,96], and contributions of unique insight into the daily lives of patients [116]. Over time, care delivered with support of HWT, such as telehealth consultations, can result in trustful relationships [116]. The introduction of technologies has the potential to alter the dynamic of relationships between patients, families, and community PC clinicians, serving as a means to complement in-person care [119]. Among the included studies, there were also reports of positive impacts on patient–caregiver communication regarding symptom management [66] and perceptions that it was easier to discuss psychological and care needs remotely [120].

Moreover, HWT can be used to facilitate companionship with and among significant others and informal caregivers [67,78,104,121-125]. The use of technology, such as video calls, was shown to be able to connect patients and family members at the end of life [78]. Another area of use is online support groups for informal caregivers, with the aim of forming companionship between peers [121].

Other studies have shown the ability of HWT to increase professional collaboration [126,127], offering an understanding of each other’s professions [126].

#### Facilitating a Comprehensive Support System

This theme focuses on the ability to use HWT to support professionals’ understanding, patient empowerment, and support for the caring role among informal caregivers. Telehealth consultation and conference technology [56,57,67,91,96,102,103,110-112,114,117,118,127,129,133] and technology for education and support [56,86,125,126,128,130-135,137-139] were the most common HWT, followed by technology for symptom monitoring [96,110,117,138,139] and remote therapy and treatment interventions [62].

Among the included studies, HWT was used to support professionals’ understanding. Oelschlägel et al [110] described how remote home care helped municipal health care professionals shift their perspective toward patients’ priorities. In the study by Rosa et al [118], remote care used as an alternative to in-person care during the COVID-19 pandemic led to changes in perceived self-efficacy among health care professionals in managing their job responsibilities. HWT, such as videoconferences, can also be used by senior professionals to support and mentor the junior workforce [86].

According to the included studies, HWT can play a role in empowering patients, for example by increasing involvement and enabling patients to take an active part in their care [96,117], feel in control of treatment [117], and manage everyday life [110]. HWT was useful in preparing patients before care visits [128], providing support [91,102,112,129], facilitating comfort, safety, and independence for patients [103,114], and increasing patient satisfaction [68].

Technologies were also used to support informal caregivers. Examples of technologies addressing their needs included interventions for mental and emotional support [63,130,131]. These were shown to have potential for preparing individuals for the caring role [86] and addressing unmet needs [63]. Digital technology interventions were used to support informal caregivers in managing everyday challenges and stress [57,132-135]. For example, an education intervention delivered through a smartphone app was effective in improving family readiness and quality of life among family members [135]. However, other studies were not able to show this potential. According to Dionne-Odom et al [130] a telehealth intervention to educate and support informal caregivers of patients with heart failure did not provide any significant effects on quality of life, mood, or caregiver burden.

Other technologies, which primarily focus on patient’s needs, may also be used to support the caregiver role, with increased caregiver involvement [136], increased connection between caregivers and patients [125], reduced caregiver burden [64,137], and effects such as less negative mood and emotional distress [138], as well as reduced loneliness [134]. Access to hospice personnel provided by HWT might be perceived as comforting [68]. Among the included studies, one study was not able to show any significant effects on caregiver burden, self-efficacy, or quality of life with the use of an eHealth self-management application for caregivers of patients with incurable cancer [139].

#### Organizational Outcomes

Organizational outcomes reported were related to cost-effectiveness [61,111], time and travel [96,108,109,114,140], and job satisfaction and distress [62,68,111,118]. The most common HWT related to the theme was telehealth consultations and conferences [61,62,68,96,108,109,111,114,118,140], although one study also involved HWT for symptom monitoring [96].

Two studies reported results related to cost-effectiveness. Compared with traditional in-person service, telehealth services resulted in equivalent costs but greater efficiency by allowing PC to reach more patients [111]. Video consultations with the PC team for rural patients were found feasible and resulted in travel and cost savings for patients [62]. The potential to minimize travel is clear, as reported by several studies. According to the case report by Morgan et al [96], telehealth-supported care was an effective adjunct to routine clinical care, Groothuizen [109] reported that virtual PC team meetings reduced travel time. Others reported that video-based consultations reduced the burden and expense of travel for patients, families, and consultants [108,114], and patient-reported satisfaction with telehealth in oncology was mainly attributed to advantages in travel and time savings [140]. Using telehealth services and eHealth systems increased job satisfaction due to the patient-centered nature of the care service, increased peer support, and increased professional development [111]; having 24/7 access to hospice triage personnel [68]; and effective care and good patient experiences [58]. On the other hand, a multidisciplinary PC team delivering telecare for hospitalized patients with cancer during the COVID-19 pandemic expressed distress related to competing loyalties (such as institutional obligations, ethical obligations to patients, resentment, and distrust of leadership) and feelings of disempowerment (due to guilt in providing subpar support, decisional regret, and loss of identity as a provider) [118].

## Discussion

### Principal Results

According to the findings, HWT has the potential to facilitate interventions to support symptom management. Different HWT interventions have shown usefulness to increase knowledge and understanding of health, diagnosis, symptoms, and concerns among patients, informal caregivers, and health care professionals. There were a variety among the reports regarding impact on quality of life among patients and caregivers; although, HWT can sometimes be useful to support dying persons and family members to bring closure and reconciliation. Related to PC provision, the use of HWT has shown promising results on care satisfaction and on medication use and compliance with therapies. Technology has also been used to accelerate advance care planning, with promising results and positive effects on the use of emergency and hospital care. One large benefit is the increased access to care using HWT, which means comfort and security for patients and families. HWT can be useful to include and involve significant others. On the other hand, research has shown both positive and negative impacts on the quality of partnership between patients, families, and health care teams when using HWT, suggesting situations when physical meetings are preferred and others when online meetings are suitable to build and maintain good quality care. From an organizational perspective, HWT has the potential to save time and costs due to decreased travelling by using digital means to consultations. The use of digital tools can increase job satisfaction but also contribute to perceived job strains and job distress.

### Comparison With Prior Work

PC is a holistic approach to care for people with life-limiting conditions and near end of life. It aims to acknowledge and attend to all aspects of the patients’ and family members’ needs, including physiological, psychological, existential, and social issues [3]. Symptom management is identified as one of the critical areas in PC [141]. According to the current review, HWT can have a positive impact on physical and mental symptoms and support symptom management. Patients in PC suffer from advanced and chronic conditions, often with a negative progression. There was some evidence for the potential of using remote monitoring and care support to prevent disease progression or functional decline, at least for some time. Not very surprisingly, there were no studies presenting any significant effects on mortality.

For symptom management, timely and close contact with the health care team is essential [141]. It has long been recognized that access to and communication with the PC team are vitally important for patients as well as informal caregivers [142,143]. Based on the findings, HWT provides new ways of access and continuity of care, with improved opportunities to follow patients’ needs without travelling. The support of patients to remain and continue to receive care at home [31,32] is especially important in cases when clinical conditions or geographic location prevent patients from accessing conventional care [33].

Although there is a concern that the partnership between patients, informal caregivers, and the PC team might be suffering. According to included studies, digital means of communication could be perceived as difficult due to loss of nonverbal cues and limited proximity to the patient when not being in the same room. Meeting online instead of in person can affect the possibilities for the health care professional to be supportive and comforting during difficult situations. Other reviews, on the other hand, have concluded that technology has the potential to not only facilitate communication through the inherent flexibility provided by technology [35] but also to build and enhance relationships [30,31]. These contradictory findings point toward the importance of caution in choosing when to use HWT in PC and for what patients.

Informal caregivers have an important role and take great responsibility for everyday symptom and medicine management [143]. As having insight into the patient’s well-being and needs, they are an important link between patient and the care team, relaying concerns, managing medication, and generally advocating for the patient when they are unable or unwilling to do so [143]. However, they also carry a heavy burden, while they must manage their own health and grief. It is, therefore, vital that they are adequately supported. Among the included studies, there was HWT addressing the caring role, aimed at supporting informal caregivers. Moreover, HWT focusing on the patients’ needs were also shown to have an impact on the situation for informal caregivers. PC should take a holistic view of the patient and the informal caregiver, the concerns of both being intertwined and interdependent [143]. Based on the findings, there is research supporting the potential for HWT to complement the ordinary PC to increase support for the caregiving role.

Besides symptom management, access to the health care team, and support of informal caregivers, interprofessional teamwork is emphasized as a crucial element in PC [3]. From a professional perspective, the use of HWT means both possibilities and challenges. According to the included studies, the use of HWT may increase the possibility for professionals to reach the patients and their families when needed, for example, to follow progression and adjust treatment. On the other hand, according to some of the studies, this means yet another responsibility, which may contribute to increased job strain among health care professionals. Previous research suggests that HWT can improve the coordination of care and help build and enhance personal and professional relationships [30]. Other potential effects are time saving and reduction of no-show rates [27].

### Limitations and Future Directions

The methodology of this study involved a comprehensive, state-of-the-art review of the existing literature on HWT in PC. With the purpose of enhancing reliability and contributing to the generalizability of findings, the search was conducted in a variety of electronic databases—APA, PsycINFO, Cochrane Library, CINAHL Plus, PubMed, Scopus, and Web of Science Core Collection. A supplementary search ensured up-to-date coverage and inclusion of recent research developments in the rapidly evolving field of HWT. In the search, several concepts were used to describe technology-based interventions in health care, often used interchangeably [144]. HWT is a common term in Nordic countries, but not as commonly used elsewhere. To ensure broad inclusion of relevant studies, broader terms like mHealth, telemedicine, and ambient intelligence were used, although specific technologies such as alarms or monitoring were avoided [145]. This approach may have limited our results to more general articles, excluding those focused on specific technologies.

Notably, among the included studies, there was a predominance of studies focusing on patients with cancer, often neglecting other palliative conditions. Furthermore, the limited diversity in participant demographics—with studies often focused on specific age groups, genders, or cultural contexts—highlights a gap in the representativeness of the findings. Future research should aim to include a wider range of diagnoses and settings to ensure that the findings are applicable to the broader PC population [36]. Moreover, there is a need for more rigorous research related to patient outcomes and evidence regarding effectiveness [29], health system outcomes (eg, usage and costs) [146], and best practices for quality remote PC [27]. New literature reviews should be conducted in a few years to update the state of the art.

### Conclusions

PC is both a clinical specialty and an overall approach to care that focuses on improving quality of life and relieving suffering for patients and families facing serious illnesses, based on need and not prognosis. HWT shows potential as a complement to usual PC to increase access and continuity of care, for symptom management, to support patients to remain at home and prevent frequent emergency visits. In some situations, it has the potential to build and maintain relationships between patients, their families, and the health care team, as well as serve as a means for increased interprofessional collaboration and support. However, there are challenges to overcome that might affect the quality of care using HWT. It is unclear whether PC delivered remotely or with support of HWT is equivalent to the more resource-intensive in-person care. Our findings point toward the importance of caution in choosing when to use HWT in PC and for which patients.

## Appendix

Multimedia Appendix 1 PRISMA-ScR checklist.

Multimedia Appendix 2 Documentation database searches.

Multimedia Appendix 3 Characteristics of included studies.

## Abbreviations

COPD: chronic obstructive pulmonary disease
HWT: health and welfare technology
MeSH: Medical Subject Headings
PC: palliative care
PRISMA: Preferred Reporting Items for Systematic Reviews and Meta-Analyses
PRISMA-ScR: Preferred Reporting Items for Systematic Reviews and Meta-Analyses extension for Scoping Reviews

## Data Availability

All data generated or analyzed during this study are included in this published article and its supplementary information files.
