# Supplementary material for: Use of Health and Welfare Technology in Palliative Care: State-of-the-Art Review
Source: J Med Internet Res. 2026 Mar 12;28:e79637. doi: 10.2196/79637 (PMC13022555; doi:10.2196/79637)
Supplement: Multimedia Appendix 2 [file jmir_v28i1e79637_app2.docx]

### Multimedia Appendix 2. Documentation of search strategies: PubMed, APA PsycINFO, Cochrane Library, Cinahl Plus, Scopus, and Web of Science Core Collection

### PubMed ([www.ncbi.nlm.nih.gov/pubmed](http://www.ncbi.nlm.nih.gov/pubmed))

|  |  |
| --- | --- |
| **Search #** | *Search words* |
|  | "Palliative Care"[MeSH Terms] OR "Palliative Medicine"[MeSH Terms] OR "Hospice and Palliative Care Nursing"[MeSH Terms] OR "Terminally ill"[MeSH Terms] OR "Terminal Care"[MeSH Major Topic] OR "Hospice Care"[MeSH Terms] |
|  | "palliati*"[Title/Abstract] OR "hospice care"[Title/Abstract] OR "hospice nursing"[Title/Abstract] OR "terminal care"[Title/Abstract] OR "supportive care"[Title/Abstract] OR "terminal stage"[Title/Abstract] OR "terminal disease"[Title/Abstract] OR "terminally ill"[Title/Abstract] OR "end stage"[Title/Abstract] OR "end of life"[Title/Abstract] OR "hospice program*"[Title/Abstract] OR "advanced illness"[Title/Abstract] |
|  | #1 OR #2 |
|  | "eHealth"[Title/Abstract] OR "e-health"[Title/Abstract] OR "telemedicine"[Title/Abstract] OR "telehealth"[Title/Abstract] OR "mhealth"[Title/Abstract] OR "m-health"[Title/Abstract] OR "mobile health"[Title/Abstract] OR "e-homecare"[Title/Abstract] OR "digital health"[Title/Abstract] |
|  | "Telemedicine"[MeSH Terms] |
|  | #4 OR #5 |
|  | "welfare technolog*"[Title/Abstract] OR "ambient assisted living*"[Title/Abstract] OR "ambient intelligence*"[Title/Abstract] |
|  | "Ambient Intelligence"[MeSH Terms] |
|  | #7 OR #8 |
|  | "home monitoring"[Title/Abstract] OR "distance monitoring"[Title/Abstract] OR "distance care"[Title/Abstract] OR "distance nursing"[Title/Abstract] OR "distance medicine"[Title/Abstract] OR "environmental control*"[Title/Abstract] OR "remote sensing"[Title/Abstract] OR "Distance Counseling"[Title/Abstract] OR "Internet-Based Intervention"[Title/Abstract] OR "ambulatory monitoring"[Title/Abstract] OR "remote consultation"[Title/Abstract] OR "telecommunication*"[Title/Abstract] OR "E-Counseling"[Title/Abstract] OR "e therapy"[Title/Abstract] OR "distance spanning"[Title/Abstract] OR "health informatics"[Title/Abstract] OR "health information technology"[Title/Abstract] OR "medical information science"[Title/Abstract] |
|  | "Remote Sensing Technology"[MeSH Terms] OR "Internet-Based Intervention"[MeSH Terms] OR "monitoring, ambulatory"[MeSH Terms] OR "Remote Consultation"[MeSH Terms] OR "Telecommunications"[MeSH Terms] |
|  | #10 OR #11 |
|  | #6 OR #9 OR #12 |
|  | #3 AND #13 |
|  | "infant*"[Text Word] OR "neonat*"[Text Word] OR "newborn*"[Text Word] OR "pediatr*"[Text Word] OR "paediatr*"[Text Word] |
|  | #14 NOT #15 |
| Limits | Filters applied: from 2012/1/1 - 3000/12/12. |
|  |  |
| **Total** | **2023-11-23 = 1,455** |
|  | **2022-10-26 = 1, 226** |

### APA PsycInfo (EBSCOhost)

|  |  |
| --- | --- |
| **Search #** | *Search words* |
|  | (DE "Palliative Care") OR (DE "Terminally Ill Patients") OR (DE "Hospice") |
|  | (TI palliati* OR AB palliati*) OR (TI "hospice care" OR AB "hospice care") OR (TI "hospice nursing" OR AB "hospice nursing") OR (TI "terminal care" OR AB "terminal care") OR (TI "supportive care" OR AB "supportive care") OR (TI "terminal stage" OR AB "terminal stage") OR (TI "terminal disease" OR AB "terminal disease") OR (TI "terminally ill" OR AB "terminally ill") OR (TI "end stage" OR AB "end stage") OR (TI "end of life" OR AB "end of life") OR (TI "hospice program*" OR AB "hospice program*") OR (TI "advanced illness" OR AB "advanced illness") |
|  | #1 OR #2 |
|  | (TI eHealth OR AB eHealth) OR (TI e-health OR AB e-health) OR (TI telemedicine OR AB telemedicine) OR (TI telehealth OR AB telehealth) OR (TI mhealth OR AB mhealth) OR (TI m-health OR AB m-health) OR (TI "mobile health" OR AB "mobile health") OR (TI e-homecare OR AB e-homecare) OR (TI "digital health" OR AB "digital health") |
|  | (DE "Telemedicine") OR (DE "Mobile Health") |
|  | #4 OR #5 |
|  | (TI "welfare technolog*" OR AB "welfare technolog*") OR (TI "ambient assisted living*" OR AB "ambient assisted living*") OR (TI "ambient intelligence*" OR AB "ambient intelligence*") |
|  | (TI "home monitoring" OR AB "home monitoring") OR (TI "distance monitoring" OR AB "distance monitoring") OR (TI "distance care" OR AB "distance care") OR (TI "distance nursing" OR AB "distance nursing") OR (TI "distance medicine" OR AB "distance medicine") OR (TI "environmental control*" OR AB "environmental control*") OR (TI "remote sensing" OR AB "remote sensing") OR (TI "Distance Counseling" OR AB "Distance Counseling") OR (TI "Internet-Based Intervention" OR AB "Internet-Based Intervention") OR (TI "ambulatory monitoring" OR AB "ambulatory monitoring") OR (TI "remote consultation" OR AB "remote consultation") OR (TI telecommunication* OR AB telecommunication*) OR (TI E-Counseling OR AB E-Counseling) OR (TI "e therapy" OR AB "e therapy") OR (TI "distance spanning" OR AB "distance spanning") OR (TI "health informatics" OR AB "health informatics") OR (TI "health information technology" OR AB "health information technology") OR (TI "medical information science" OR AB "medical information science") |
|  | (DE "Teleconferencing") OR (DE "Videoconferencing") OR (DE "Teleconsultation") OR (DE "Telepsychiatry") OR (DE "Telepsychology") OR (DE "Digital Interventions") OR (DE "Telerehabilitation") OR (DE "Videoconferencing") OR (DE "Online Therapy") OR (DE "Telecommunications Media") |
|  | #8 OR #9 |
|  | #6 OR #7 OR #10 |
|  | #3 AND #11 |
|  | infant* OR neonat* OR newborn* OR pediatr* OR paediatr* |
|  | #12 NOT #13 |
| Limits | Publication Year: 2012-2023; Peer Reviewed |
|  |  |
| **Total** | **2023-11-23 = 180** |
|  | **2022-10-26 = 136** |

### Cochrane Library, ([www.cochranelibrary.com](http://www.cochranelibrary.com))

|  |  |
| --- | --- |
| **Search #** | *Search words* |
|  | [mh "Palliative Care"] OR [mh "Palliative Medicine"] OR [mh "Hospice and Palliative Care Nursing"] OR [mh "Terminally ill"] OR [mh "Terminal Care"] OR [mh "Hospice Care"] |
|  | palliati*:ti,ab OR "hospice care":ti,ab OR "hospice nursing":ti,ab OR "terminal care":ti,ab OR "supportive care":ti,ab OR "terminal stage":ti,ab OR "terminal disease":ti,ab OR "terminally ill":ti,ab OR "end stage":ti,ab OR "end of life":ti,ab OR "hospice NEXT program":ti,ab OR "advanced illness":ti,ab |
|  | #1 OR #2 |
|  | eHealth:ti,ab OR e-health:ti,ab OR telemedicine:ti,ab OR telehealth:ti,ab OR mhealth:ti,ab OR m-health:ti,ab OR "mobile health":ti,ab OR e-homecare:ti,ab OR "digital health":ti,ab |
|  | [mh Telemedicine] |
|  | #4 OR #5 |
|  | "welfare NEXT technolog":ti,ab OR "ambient assisted living":ti,ab OR "ambient NEXT intelligence":ti,ab |
|  | [mh "Ambient Intelligence"] |
|  | #7 OR #8 |
|  | "home monitoring":ti,ab OR "distance monitoring":ti,ab OR "distance care":ti,ab OR "distance nursing":ti,ab OR "distance medicine":ti,ab OR "environmental NEXT control":ti,ab OR "remote sensing":ti,ab OR "Distance Counseling":ti,ab OR "Internet-Based Intervention":ti,ab OR "ambulatory monitoring":ti,ab OR "remote consultation":ti,ab OR telecommunication*:ti,ab OR E-Counseling:ti,ab OR "e therapy":ti,ab OR "distance spanning":ti,ab OR "health informatics":ti,ab OR "health information technology":ti,ab OR "medical information science":ti,ab |
|  | [mh "Remote Sensing Technology"] OR [mh "Internet-Based Intervention"] OR [mh "monitoring, ambulatory"] OR [mh "Remote Consultation"] OR [mh Telecommunications] |
|  | #10 OR #11 |
|  | #6 OR #9 OR #12 |
|  | #3 AND #13 |
|  | infant*:ti,ab,kw OR neonat*:ti,ab,kw OR newborn*:ti,ab,kw OR pediatr*:ti,ab,kw OR paediatr*:ti,ab,kw |
|  | #14 NOT #15 |
| Limits | 2012-2022 |
|  |  |
| **Total** | **2023-11-23 = 316 (2 Cochrane reviews and 314 Trials)**  **2022-10-26 = 275 (2 Cochrane reviews och 273 Trials)** |

### Cinahl Plus (EBSCOhost)

|  |  |
| --- | --- |
| **Search #** | *Search words* |
|  | (MH "Palliative Medicine") OR (MH "Hospice and Palliative Nursing") OR (MH "Palliative Care") OR (MH "Terminally Ill Patients+") OR (MH "Hospice Care") OR (MH "Hospice Patients") |
|  | (TI palliati* OR AB palliati*) OR (TI "hospice care" OR AB "hospice care") OR (TI "hospice nursing" OR AB "hospice nursing") OR (TI "terminal care" OR AB "terminal care") OR (TI "supportive care" OR AB "supportive care") OR (TI "terminal stage" OR AB "terminal stage") OR (TI "terminal disease" OR AB "terminal disease") OR (TI "terminally ill" OR AB "terminally ill") OR (TI "end stage" OR AB "end stage") OR (TI "end of life" OR AB "end of life") OR (TI "hospice program*" OR AB "hospice program*") OR (TI "advanced illness" OR AB "advanced illness") |
|  | #1 OR #2 |
|  | (TI eHealth OR AB eHealth) OR (TI e-health OR AB e-health) OR (TI telemedicine OR AB telemedicine) OR (TI telehealth OR AB telehealth) OR (TI mhealth OR AB mhealth) OR (TI m-health OR AB m-health) OR (TI "mobile health" OR AB "mobile health") OR (TI e-homecare OR AB e-homecare) OR (TI "digital health" OR AB "digital health") |
|  | (MH "Telehealth+") |
|  | #4 OR #5 |
|  | (TI "welfare technolog*" OR AB "welfare technolog*") OR (TI "ambient assisted living*" OR AB "ambient assisted living*") OR (TI "ambient intelligence*" OR AB "ambient intelligence*") |
|  | (TI "home monitoring" OR AB "home monitoring") OR (TI "distance monitoring" OR AB "distance monitoring") OR (TI "distance care" OR AB "distance care") OR (TI "distance nursing" OR AB "distance nursing") OR (TI "distance medicine" OR AB "distance medicine") OR (TI "environmental control*" OR AB "environmental control*") OR (TI "remote sensing" OR AB "remote sensing") OR (TI "Distance Counseling" OR AB "Distance Counseling") OR (TI "Internet-Based Intervention" OR AB "Internet-Based Intervention") OR (TI "ambulatory monitoring" OR AB "ambulatory monitoring") OR (TI "remote consultation" OR AB "remote consultation") OR (TI telecommunication* OR AB telecommunication*) OR (TI E-Counseling OR AB E-Counseling) OR (TI "e therapy" OR AB "e therapy") OR (TI "distance spanning" OR AB "distance spanning") OR (TI "health informatics" OR AB "health informatics") OR (TI "health information technology" OR AB "health information technology") OR (TI "medical information science" OR AB "medical information science") |
|  | (MH "Remote Consultation") OR (MH "Internet-Based Intervention") OR (MH "Telemetry") OR (MH "Telecommunications+") |
|  | #8 OR #9 |
|  | #6 OR #7 OR #10 |
|  | #3 AND #11 |
|  | infant* OR neonat* OR newborn* OR pediatr* OR paediatr* |
|  | #12 NOT #13 |
| Limits | Publication Year: 2012-; Peer Reviewed |
|  |  |
| **Total** | **2023-11-23 = 1,246**  **2022-10-26 = 1,098** |

### Scopus, ([www.scopus.com](http://www.scopus.com))

|  |  |
| --- | --- |
| **Search #** | *Search words* |
|  | TITLE-ABS-KEY ( palliati* OR "hospice care" OR "hospice nursing" OR "terminal care" OR "supportive care" OR "terminal stage" OR "terminal disease" OR "terminally ill" OR "end stage" OR "end of life" OR "hospice program*" OR "advanced illness" ) |
|  | TITLE-ABS-KEY ( ehealth OR e-health OR telemedicine OR telehealth OR mhealth OR m-health OR "mobile health" OR e-homecare OR "digital health" ) |
|  | TITLE-ABS-KEY ( "welfare technolog*" OR "ambient assisted living*" OR "ambient intelligence*" ) |
|  | TITLE-ABS-KEY ( "home monitoring" OR "distance monitoring" OR "distance care" OR "distance nursing" OR "distance medicine" OR "environmental control*" OR "remote sensing" OR "Distance Counseling" OR "Internet-Based Intervention" OR "ambulatory monitoring" OR "remote consultation" OR telecommunication* OR e-counseling OR "e therapy" OR "distance spanning" OR "health informatics" OR "health information technology" OR "medical information science" ) |
|  | #2 OR #3 OR #4 |
|  | #1 AND #5 |
|  | TITLE-ABS-KEY ( infant* OR neonat* OR newborn* OR pediatr* OR paediatr* ) |
|  | #6 NOT #7 |
| Limits | ( LIMIT-TO ( PUBYEAR , 2022 ) OR LIMIT-TO ( PUBYEAR , 2021 ) OR LIMIT-TO ( PUBYEAR , 2020 ) OR LIMIT-TO ( PUBYEAR , 2019 ) OR LIMIT-TO ( PUBYEAR , 2018 ) OR LIMIT-TO ( PUBYEAR , 2017 ) OR LIMIT-TO ( PUBYEAR , 2016 ) OR LIMIT-TO ( PUBYEAR , 2015 ) OR LIMIT-TO ( PUBYEAR , 2014 ) OR LIMIT-TO ( PUBYEAR , 2013 ) OR LIMIT-TO ( PUBYEAR , 2012 ) OR LIMIT-TO ( PUBYEAR , 2023 ) OR LIMIT-TO ( PUBYEAR , 2024 ) ) |
|  |  |
| **Total** | **2023-11-23 = 2,042 2022-10-26 = 1,674** |

### Web of Science, ([www.webofscience.com](http://www.webofscience.com))

|  |  |
| --- | --- |
| **Search #** | *Search words* |
|  | palliati* OR "hospice care" OR "hospice nursing" OR "terminal care" OR "supportive care" OR "terminal stage" OR "terminal disease" OR "terminally ill" OR "end stage" OR "end of life" OR "hospice program*" OR "advanced illness" (Topic) |
|  | eHealth OR e-health OR telemedicine OR telehealth OR mhealth OR m-health OR "mobile health" OR e-homecare OR "digital health" (Topic) |
|  | "welfare technolog*" OR "ambient assisted living*" OR "ambient intelligence*" (Topic) |
|  | "home monitoring" OR "distance monitoring" OR "distance care" OR "distance nursing" OR "distance medicine" OR "environmental control*" OR "remote sensing" OR "Distance Counseling" OR "Internet-Based Intervention" OR "ambulatory monitoring" OR "remote consultation" OR telecommunication* OR E-Counseling OR "e therapy" OR "distance spanning" OR "health informatics" OR "health information technology" OR "medical information science" (Topic) |
|  | #2 OR #3 OR #4 |
|  | #1 AND #5 |
|  | infant* OR neonat* OR newborn* OR pediatr* OR paediatr* (Topic) |
|  | #6 NOT #7 |
| Limits | Refined By: Publication Years: 2012 or 2013 or 2014 or 2015 or 2016 or 2017 or 2018 or 2019 or 2020 or 2021 or 2022 or 2023 |
|  |  |
| **Total** | **2023-11-23 = 1,153**  **2022-10-26 = 948** |
