# Supplementary material for: Use of Health and Welfare Technology in Palliative Care: State-of-the-Art Review
Source: J Med Internet Res. 2026 Mar 12;28:e79637. doi: 10.2196/79637 (PMC13022555; doi:10.2196/79637)
Supplement: Multimedia Appendix 3 [file jmir_v28i1e79637_app3.docx]

Multimedia Appendix 3. Characteristics of included studies.

| **Study** | **Country** | **Design** | **Target group** | **HWT** | **Findings** |
| --- | --- | --- | --- | --- | --- |
| Alcalde Castro et al., 2023 | Canada | Qualitative grounded theory study | Patients at outpatient palliative care oncology clinic. | Telehealth visits | Participants endorsed a flexible hybrid approach incorporating both in-person and telehealth visits. Specific categories were: (1) in-person outpatient palliative care supported building interpersonal connections and trust; (2) telehealth palliative care facilitated greater efficiency, comfort and independence and (3) patient-preferred circumstances for in-person visits (preferred for initial consultations, visits where a physical examination may be required and advance care planning discussions), versus telehealth visits (preferred during periods of relative heath stability). |
| Applebaum et al., 2018 | USA | Randomized control trial | Cancer caregivers | Self‐administered web‐based program, the Care for the Cancer Caregiver (CCC), psycho-therapeutic intervention. | some observed mean change scores and effect sizes were consistent with hypothesized trends (eg, meaning in caregiving, benefit finding, and depressive symptomatology), though no pre‐post significant differences emerged between groups. However, a longitudinal mixed‐effects model found significant differential increases in benefit finding in favor of the CCC arm. |
| Ariss et al., 2021 | United Kingdom | Mixed-methods, theory-driven evaluation | Professionals in palliative care services | Digital health technology designed to provide remote, real-time support and task delegation | The digital health intervention allowed the service to include a more junior workforce requiring fewer joint visits. No negative changes in hospitalization were observed and patient reported experiences improved. Changes in hospital non-emergency bed days were inconclusive. However, emergency department admissions reduced significantly. The cost per hour for visits reduced and annual savings of £135153 are estimated for reduced emergency admissions. |
| Atreya et al., 2020 | India | Exploratory survey design | Professionals in advanced cancer care | Teleconsultation service with a triage system | Although there was no change in the number of emergency visits, the inpatient admissions reduced by 44%. Nearly 82% of patient/caregivers gave a positive feedback about telemedicine care provided by the department and mentioned that the service provided them with support and connectedness. Almost 64% of the patients and caregivers reported that the service helped allay the fear and reassured them that there was someone to support them. As high as 76% of the participants felt that they would prefer teleconsultation in future and were ready to pay for teleconsultations if charges were to be applied in the future. |
| Baird-Bower et al., 2016 | Australia | Retrospective study | Palliative care patients | A state-wide palliative care after-hours number called 1800HOSPICE | Family and caregivers mainly used the after-hours support to request changes to support services, report changes in patients’ overall condition and request acute medical assistance. Through the use of the after-hours services by all individuals involved in the care, end-of-life patients were able to reduce ambulance contact and emergency department admission, and thereby increase their likelihood of dying at home (for patients whose preference was to die at home). |
| Bandini et al., 2022 | USA | Mixed methods comparative study | Outpatient palliative care patients | Telehealth for outpatient palliative care | Patients in both groups reported highly positive experiences; there were no differences in “feeling heard and understood” or the overall ratings of the provider and team between the telehealth-only and in-person-only groups. Palliative care program leaders described the benefits and challenges of telehealth, including increased efficiency, the ability to incorporate family members, and challenges conducting a physical examination. |
| Bange et al., 2023 | USA | Retrospective cohort study | Patients with incurable or high-risk cancer | Telemedicine (video or phone) | The occurrence of advance care planning was similar between telemedicine and in‐person visits. In regard to telemedicine subtype, patients exposed to video encounters were modestly more likely to have documented advance care planning in comparison to those seen in person. The 3‐month risk for unplanned hospitalization was comparable for telemedicine compared to in‐person clinic encounters. |
| Baxter et al., 2021 | USA | Quality improvement pilot study | Older adults in nursing homes | Palliative telehealth program including a palliative care consultant | The pilot shows promising outcomes including code status change, Medical Order for Life-Sustaining Treatment form completion, and reduced hospitalizations supporting the benefit of comprehensive goals-of-care discussions for frail older adults living in a nursing home. |
| Beesley et al., 2020 | Australia | Single-arm feasibility study | Patients with pancreatic cancer and caregivers | PREPARES (Patients and RElatives affected by PAncreatic cancer: Referral, Education and Support) pilot intervention. Counselling sessions delivered by a trained nurse via telephone and/or telehealth technology. | The intervention was highly acceptable. Participants unanimously preferred the telephone over video-conferencing and to receive counselling separately from their carer/patient. The main perceived benefits were emotional support, the nurse-counsellors’ knowledge, care coordination and personalized care. |
| Benson, et al., 2020 | USA | Mixed methods study | Family caregivers of palliative cancer patients | Facebook support group with a social worker acting as facilitator. | Analyses demonstrated that the majority of online support provided by group members was emotional support, followed by companionship support, appraisal support, and informational support. Instrumental support was rarely provided. Support was primarily elicited in an indirect manner through self-disclosure and patient updates, with few overt requests for support. |
| Bentley et al., 2020 | Australia | Pilot study | Patients with terminal illness | Dignity therapy delivered by two therapist researchers via videoconference or e-mail. | Participants reported high levels of acceptability and efficacy comparable to face-to-face delivery; meanwhile therapist time was about 40% less and legacy documents were longer. Participants described dignity therapy online as convenient, but technological issues may create challenges. |
| Besse et al., 2016 | Netherlands | Feasibility study | Palliative care patients with cancer | Daily IVR/SMS to patients asking to provide their pain score on a numeric rating scale (NRS) with their mobile phone. | IVR/SMS provides a reliable assessment of the pain intensity, and if required, treatment can be rapidly adapted. All patients were satisfied with the intervention. There were no difficulties for the, mainly older, patients in handling this communication way on pain intensity. Overall pain score decreased. |
| Bhargava et al., 2021 | Canada | Pilot feasibility study | Patients with palliative care needs | RELIEF, a remote symptom self-reporting app for community patients with palliative care needs. | Patients completed 80% of daily self-report assessments; 133 alerts were trigged, half of which required clinical intervention. No patient visited the emergency department for symptom management during the study. Clinical staff estimated five emergency department visits were avoided because of RELIEF—saving an estimated cost of over CAD 60,000. |
| Bonsignore et al., 2018 | USA | Mixed methods feasibility study | Patients in rural palliative care | A telehealth community-based palliative care program using remote patient monitoring via the TapCloud application and videoconferencing | Remote patient monitoring using TapCloud resulted in improved symptom management. Patients, caregivers, and providers reported positive experiences with telehealth with three main advantages: 1) access to clinicians, 2) quick responses, and 3) improved efficiency and quality of care. |
| Bückmann et al., 2023 | Germany | Multi-center feasibility trial | Health care professionals | Telemedical consultations to transfer expertise between inpatient tertiary and primary care hospitals | Overall, teleconsultations were considered helpful in resolving issues for 97.9% of the cases. Yet, technical problems arose in about one-third of all meetings for at least one physician. Telemedicine has the potential to transfer university expertise to external hospitals through simple means. It improves collaboration among physicians, may prevent unnecessary transfers or outpatient presentations, and is thus likely to lower costs. |
| Cameron, 2021 | USA | Mixed-methods study | Caregivers of home hospice patients | Tele-hospice program including an electronic tablet with the AVA application (Angela’s Virtual Assist) | Most respondents reported being comfortable using telehospice, and as comfort levels increased, caregivers were more willing to use virtual healthcare again. Positive, negative, and neutral themes emerged from the comments by caregivers. The results of this study provide support for using telehospice with caregivers of home hospice patients. Caring for a person at the end of life is stressful and having the ability to contact a professional 24 hours a day provided comfort to the caregivers. Training is essential for caregivers to use technology and integration of technology in the care plans for all hospice patients is important for success. |
| Chih et al., 2013 | USA | Randomized trial | Caregivers of patients with advanced-stage lung, breast, and prostate cancer | Comprehensive Health Enhancement Support System, an interactive cancer communication system. | Caregivers in the Comprehensive Health Enhancement Support System + Clinician Report group reported less negative mood than those in the Comprehensive Health Enhancement Support System-Only group at both 6 months and 12 months. Groups were not significantly different on caregiver preparedness or physical burden at either time point. |
| Chiu wu et al., 2020 | Taiwan | Cluster-randomized controlled trial | Community-dwelling older adults | A multimedia educational intervention followed  by telephone consultation | The study showed that 100% of participants in the intervention group selected hospice program care for end-of-life care and signed Advanced Directives, whereas those in the control group were less likely to do both. Participants in the intervention group also had a positive change in knowledge, subjective norms, perceived behavioral control, and behavioral intention of advance care planning for advanced dementia. |
| Collier et al., 2016 | Australia | Qualitative study | Clinicians | Self-report assessment tools for patients and carers and remote activity monitoring; ongoing video-based conferences between service staff, patient or carer; and virtual case conferences with patient and carer, service staff and general practitioners. | Service providers consider telehealth resources to augment current service provision in a complementary way rather than as a replacement for face-to-face assessments. Introducing this technology, however, challenged the team to critically explore aspects of current service provision. The introduction of technologies also has the potential to alter the dynamic of relationships between patients and families and community palliative care clinicians. |
| Cornetta et al., 2023 | Kenya | Feasiblity study | Patients with advanced cancer | Telephone monitoring (Telehospice) in providing symptom management | 100% participation in weekly calls was obtained. A secondary objective was the use of “comfort kits” which contained 30 doses of six medications. Most patients utilized one or more of the provided medications, with high usage of bisacodyl, paracetamol, and omeprazole. While 12% of weekly calls and 24% of hotline calls led to medication changes, participants continued to express worry and there was only a modest decrease in pain scores despite having morphine available throughout the follow-up period. Family confidence in providing care and access to information remained high. At the end of the eight-weeks of observation, eight participants were alive, 10 died at home, and 12 were admitted to an in-patient facility. |
| Cruz-Oliver et al., 2022 | USA | Qualitative interview study | Family caregivers of people with advanced cancer | Telenovela Educational Intervention | Three unique themes were identified: acceptability of NOVELA, usability and relevance of NOVELA, and the effect of NOVELA. According to our conceptual model, the intervention positively affects all three adult learning categories: reaction, learning, and behavior. |
| Dionne-Odom et al, 2020 | United Kingdom | Randomized clinical trial | Caregivers of persons with advanced heart failure | Psychosocial and problem-solving support with telehealth | The participants did not demonstrate clinically better quality of life, mood, or burden compared with usual care over 16 weeks. |
| DuBenske et al., 2014 | USA | Randomized controlled study | Informal caregivers of patients with cancer | Internet and CHESS (Comprehensive Health Enhancement Support System, a web-based lung cancer information) | Caregivers randomized to CHESS reported lower burden and negative mood than those in the Internet group. The effect on disruptiveness was not significant. |
| Elma et al., 2022 | Canada | Qualitative descriptive study | Clinicians | Videoconferencing technology | Clinicians used videoconferencing technology to try to bridge gaps in end-of-life care by facilitating connections with family. Many benefits ensued, but there were also some drawbacks. Despite the opportunity for connection offered by virtual visits, participants noted concerns about equitable access to videoconferencing technology and authenticity of technology-assisted interactions. Participants also offered recommendations for future use of videoconferencing technology both during and beyond the pandemic. |
| Finucane et al., 2020 | United Kingdom | Mixed-methods study | Persons with an  advanced progressive illness | Electronic palliative  care coordination system, the Key Information Summary (KIS), to enable sharing of patient information, preferences across care settings, and inform emergency and out-of-hours care. | Of those with an advanced progressive illness, 69% had a KIS. These were  started a median of 45 weeks before death.  People with cancer were most likely to have a  KIS (80%), and those with organ failure least likely (47%). Overall, 68% of KIS included resuscitation status and 55%  preferred place of care. People with a KIS were more likely to die in the community compared to those without one. Most KIS were considered useful/highly useful. Up-to-date free-text information within the KIS was valued highly. |
| Graven et al., 2023 | USA | Randomized controlled pilot study | Patients with heart failure | The Coping in Heart Failure (COPE-HF) Partnership telehealth intervention. | Significant improvement in total heart failure symptoms and all sub-scales were noted in the intervention group, with the largest improvement seen in heart failure symptom severity. Findings suggest that the COPE-HF Partnership telehealth intervention may decrease symptom burden by helping patients better manage symptoms. |
| Greer et al., 2019 | USA | Randomized trial | Patients with incurable cancers | Cognitive-Behavioral Therapy Mobile Application for Anxiety | Patients in both study groups reported improvements in anxiety, depression symptoms, and QOL from baseline to post-assessment, with no significant differences in any outcome measure between groups. Secondary analyses showed that, among the subgroup of patients with severe baseline anxiety, those randomized to the CBT app had greater improvements on anxiety subscales compared with the control group. |
| Groothuizen et al., 2023 | United Kingdom | Mixed methods study | Palliative team members and managers | Virtual team meetings | Increased flexibility, reduced travel time and  easier real-time access to patient information were seen as the main advantages of virtual meetings. Views regarding the impact on relational aspects and communication differed. In line with observational findings, concerns were raised in relation to IT, including having inappropriate equipment, insufficient bandwidth and an overarching theme that virtual meeting platforms provided were not fit for purpose. |
| Gustafson et al., 2013 | USA | Randomized trial | patients with non-small cell lung cancer | An eHealth system supporting palliative care for patients with non-small cell lung cancer (Comprehensive Health Enhancement Support System [CHESS]) | Results indicated that an online support system may reduce patient symptom distress. The effect on survival bears further investigation. |
| Gustafson et al., 2017 | USA | Randomized clinical trial | Cancer patients and their families | eHealth system that alerts clinicians to significant changes in the patient’s symptoms | When severe caregiver-reported symptoms were shared with clinicians, the symptoms were more likely to be subsequently reported as improved than when the symptoms were not shared with clinicians. Fewer symptom reports were completed in the group of caregivers whose reports went to clinicians than in the CHESS-Only group, perhaps because caregivers, knowing their reports might be sent to a doctor, feared they might be bothering the clinician. |
| Halton-Hernandez et al., 2023 | United Kingdom | Qualitative interview study | Terminally ill patients | Stories for Life, a UK charity that provides a free and confidential service for terminally ill patients to audio record their “life story.” | Patients reported a feeling of catharsis while telling their story as well as being able to reflect on significant life events. However, it was challenging to convey difficult emotions while also being mindful of protecting family who may listen to the recording. Although there was some uncertainty about how the recording would be perceived by listeners, leaving a voice-recorded life account was felt to be beneficial for immediate family members, as well as maintaining a meaningful connection with future generations. |
| Hamilton et al., 2019 | Australia | Retrospective audit of records | Records of cancer patients | Telehealth in radiation oncocolgy | 106 patients participated in the satisfaction survey (231 patients invited, response rate of 46%), with the overall positive response mainly attributed to advantages in travel and time savings. 54.7% of patients selected telehealth as their preference for future consultations, 34.9% indicated a mix of telehealth and in-person consultations, and only 1 patient (0.9%) indicating in-person only. |
| Hao et al., 2021 | China | Pre- and post-training evaluation | Nurses | An e-learning intervention approach using mobile terminal combined with a virtual forum and face-to-face interactions. | After a three-week intervention, there was a significant increase in knowledge and attitudes about palliative care. The implementation of training for nurses at appropriate intervals during both education and professional life is required, especially regarding the improvement in participants’ attitudes towards death. |
| Harding et al., 2021 | India, Uganda, and Zimbabwe | Pilot study using co-design principles | Community and family caregivers  of patients in palliative home-care | Mobile phone application to enable community caregivers and family caregivers to provide real time patient outcome data to the patient’s palliative care provider | Qualitative data identified: 1) high acceptability and data usage; 2) improved understanding by team members of patient symptoms and concerns; 3) a need for better feedback to caregivers, for better prioritization of patients according to need, for enhanced training and support to use the app, and for user-led recommendations for ongoing improvement. |
| Haydon et al., 2021 | Australia | Mixed-methods study | Palliative care team | The GCHHS Community Palliative Care Service offering telehealth | Thematic analysis generated an overarching theme of Increased Job Satisfaction which staff attributed to the patient-centred nature of the telepalliative care service, the increased peer support and increased professional development. Compared with the traditional in-person service, the new telehealth-supported model resulted in equivalent costs, greater efficiency by allowing palliative care to reach more patients and improved staff job satisfaction. |
| Hayek et al., 2014 | Georgia | Prospective quality improvement study | Journals and documentation of older patients with chronic conditions in outpatient clinic | Electronic medical record (EMR) reminder system of "Advanced Directives Counseling" | Seventy-six percent of charts with Advanced Directives Counsling on their problem list had documentation of an Advanced Directives. EMR-based reminders are effective in improving documentation rates of Advanced Directives. |
| Hennemann-Krause et al., 2015 | Brazil | prospective, longitudinal, qualitative, descriptive, and case series study | patients with advanced cancer | In-person consultations and weekly web conferences with multidisciplinary team | During monitoring, the team contacted the patients on 305 occasions: there were 89 consultations at the hospital, 19 in-person assistances to the family (without the patient), 77 web conferences, 38 telephone calls, 80 emails, and 2 home visits. The mean monitoring time until death was 195 +/- 175 days. Eight patients who completed the Edmonton Symptom Assessment System (ESAS) in all interviews had lower mean distress symptom scores according to web conferences than in person. |
| Ho et al., 2022 | Sub Sahara, Tanzania | Mixed-methods study | Caregivers of untreatable hospitalized cancer patients | Mobile Palliative Care Link (mPCL), a mobile/web application, aims to extend specialist access via shared care with local health workers (LHWs) | There was an overall satisfaction with mPCL, perceived as a way to stay connected with patients and support remote symptom control. Timely access to POS responses and medical records were identified as key benefits. Primary use was for clinical status communication and care coordination. Pain and other symptom progression were the most frequently reported reasons for provider–patient interactions. Usage barriers included time required to create a new clinical record, perceived need for response to non-urgent reminders or alerts, and training necessary for competent use. System-level implementation barriers included variable patient access to smartphones and SIM cards and unreliable Internet access. |
| Hoek et al., 2017 | Netherlands | Randomized clinical trial | patients with advanced cancer | weekly, prescheduled teleconsultations with specialist palliative care consultation team. | The Total Distress Score became significantly higher in the intervention group than in the control group. The adjusted anxiety scores were higher in the intervention group than in the control group. No difference was found between the groups in adjusted depression scores or in secondary outcome measures. There was a trend towards a lower proportion of informal caregivers with a high perceived burden in the intervention group. |
| Hutchinson et al., 2022 | USA | Mixed-methods pilot study | Seriously ill out-patients | telemedicine-delivered palliative care (tele-PC) | All patients rated tele-PC as equal to, or better than, in-person PC at providing emotional support.  Identified advantages included improved access to care, reduced preparation for visits, and added value of video. Disadvantages included technical interruptions, other interruptions, and negative impact of seeing oneself. Clinicians identified several clinician strategies for recovering from technological issues, including humor. Three themes emerged regarding the emotional responsiveness of tele-PC consultations: conversation process, conversation content, and emotional support. With respect to conversation process, participants reported that tele-PC created challenges for turn-taking, use of humor, silence, interpretation of body language, eye contact and use of touch. Regarding conversation content, clinician identified several challenges: controlling the conversation, difficulty talking about death, providing emotional support, being comfortable with conversation and setting, providing empathic communication, and diminished presence. |
| Jiang et al., 2023 | Australia | prospective mixed-methods pilot study | Patients and caregivers of a community palliative-care service | A commercially available secure Web Real Time Communication (WebRTC) platform for videoconferences | Patient–caregiver feedback showed a high level of overall satisfaction. Compared to standard care, the intervention group demonstrated less functional decline from baseline at two weeks and three months after the intervention.  At 30 days prior to death, functional status remained better in the intervention group, with fewer per capita community palliative-care nursing visits, general practitioner visits and hospital admissions. The proposed  model is feasible and could be integrated successfully  into community-based palliative care. |
| Johnston et al., 2012 | Canada | Pilot study | Dying clients, their families and health professionals | Video calling via Skype | Generally, health professionals viewed video calling as an excellent means for dying persons to connect with family living at a distance, to say goodbye, and to include family members in the dying process. All family members interviewed reported being very satisfied with using video calling to connect with dying loved ones. Some clients are opposed to video calling because they don’t want family members to see them in their dying days; they wish to preserve their family’s memories of them in healthier times. Generally, clients and family members who have used video calling have been comfortable with the process. However, participants did suggest that video calling be used only when no other adequate means of connection are available. |
| Kamal et al., 2019 | USA | Randomized, active-controlled, clinical trial | Patients referred to outpatient palliative care | PCforMe, a web-based, mobile health tool that aims to educate and engage patients referred to palliative care to better prepare them for the upcoming appointment. | Preparedness for an upcoming palliative care visit increased 50% in the intervention group and 13.3% in the control group. Difference in the number of patients with improved knowledge regarding palliative care approached significance. |
| Kazankov et al., 2023 | United Kingdom | Feasibility study | Individuals with cirrhosis | the smartphone CirrhoCare® app, for daily recording of heart rate, blood pressure, weight, % body water, cognitive function. | Use of Cir-  rhoCare® is feasible, receives high patient  engagement, and allows early detection of new clinical events,  facilitating timely intervention to prevent progression of cirrhosis decompensation. |
| Kohle et al., 2017 | Netherlands | Qualitative inteview study | Partners of cancer patients | Web-based self-help intervention based on Acceptance and Commitment Therapy (ACT) and self-compassion | In general, partners appreciated the intervention, but they also expressed ambivalent feelings towards peer support, the content of the feedback of their counselor, and the ‘tunneled’ structure of the intervention. The majority of the partners reported being more self-compassionate accepting that they experienced negative thoughts and feelings, they reported that they learned to increase the distance between their thoughts and themselves, they indicated being more aware of their personal values, and they thought that they were better able to commit to those values. They also reported helpful processes such as insight and acknowledgement, positivity, the possibility to tell their story, time for themselves, and feeling closer and more connected with their partner. |
| Koufacos et al., 2023 | USA | Survey | Caregivers to veterans with dementia | Virtual webinars sessions   focused on stress management | Results suggested that stress-reduction and selfcare may be viable target areas of intervention for caregivers of veterans with dementia in both individual and group formats.  All the caregivers surveyed said that they were satisfied with the support they had received from the project. A majority (79%) of participants found the phone call sessions they participated in to be “very helpful,” while 15.8% found them to be “helpful.” All the caregivers found that the materials provided to them throughout the program to be either “very helpful” or “helpful.” All who completed the survey reported that the phone calls and mailings they received helped them at times to reduce stress. Most (82%) of the caregivers believed that specific problems they were having were improved because of this program. All the caregivers surveyed reported that they learned new things about caregiver stress and burden from the support sessions. In addition, many participants (87.5%) said that the LCSW connected them with resources or other services to help provide additional caregiver support. |
| Kuntz et al., 2020 | USA | Intervention implementation and evaluation | Palliative inpatients and their families | Telemedicine to facilitate electronic family (e-family) meetings to facilitate in-patient palliative care. | Of the family participants who agreed to be interviewed, their overall ratings of the e-family meetings were high. Over 80% of respondent families participants reported that they agreed or strongly agreed that they were able to ask all of their questions, felt comfortable expressing their thoughts and feelings with the clinical team, felt like they understood the care their loved one received, and that the virtual family meeting helped them trust the clinical team. Family respondents reported that the e-family meeting helped them understand their loved one’s thoughts and wishes. |
| LeBaron et al., 2022 | USA | Feasibility and Acceptability Study | Patients with advanced cancer and their family caregivers in rural settings | Smart health sensing system, behavioral and environmental sensing and intervention for cancer (BESI-C), designed to support monitoring and management of cancer pain in home setting | Procedural feasibility challenges included the rurality of dyads, smart watch battery life and EMA reliability, and the length of time required for deployment installation. Dyads disagreed that BESI-C was a burden or compromised their privacy and agreed that the system collected helpful information to better manage cancer pain. Participants also expressed an interest in seeing their own individual data and strongly agreed that it is important that data collected by BESI-C are shared with their respective partners and health care providers. Qualitative feedback from participants suggested that BESI-C positively improved patient-caregiver communication regarding pain management. |
| Lind & Karlsson, 2013 | Sweden | Randomized controlled study | Palliative home care patients near the end of their lives | Electronic patient-reported symptom assessment in palliative end-of-life home care | There was a significantly shorter median time span from reporting to noticing for assessments in the intervention group. The system allowed both frequent and regular symptom reporting from patients that can contribute to more correct and prompt medical decisions in palliative end-of-life home care. |
| LoCastro et al., 2023 | USA | A single-center qualitative study | Patients with hematologic malignancy, caregivers, and clinicians | The SICP, a multicomponent communication intervention developed to improve conversations about values for patients with serious illnesses for delivery via telehealth | Four qualitative themes emerged from data: 1) Serious illness conversations can be conducted via telehealth, 2) Older patients have limited experience using technology but are willing and able to learn, 3) Patients feel that serious illness conversations will help them understand their diagnosis and prognosis better, and 4) Serious illness conversations should be common and routine, not extra-ordinary. |
| Maguire et al., 2020 | United Kingdom | convergent mixed methods study | Patients with malignant pleural mesothelioma | the Advanced Symptom Management System (ASyMS), remote symptom monitoring system | There were no significant changes in quality of life. There were statistically significant improvements in the SPARC psychological need domain and in the “Usefulness” domain of the TAM. End-of-study interviews identified that both patients and clinicians found the system quick and easy to use. For patients, in particular, the system provided reassurance about symptom experience and the feeling of being listened to. The clinicians largely viewed the system as feasible and acceptable, and areas that were mentioned included the early management of symptoms and connectivity between patients and clinicians, leading to enhanced communication. |
| Manz et al., 2023 | USA | Randomized clinical trial | clinicians of patients with cancer | weekly emails to clinicians comparing their SIC rates for all patients against peers’ rates,  opt-out text messages to prompt SICs before  encounters with high-risk patients. | The intervention was associated with increased SICs for all patients and  decreased end-of-life systemic therapy  relative to controls, but there was no effect on hospice enrollment or length of stay, inpatient death, or end-of-life ICU use. |
| Marco et al., 2023 | Australia | Retrospective analysis | Callers to Palliative Care Advice Service | Telephone calls to the Palliative Care Advice Service (PCAS) | Most calls were from members of the public and related to malignant conditions. Regional/rural clients comprised 45% of  all calls to the service, of which half were health professionals seeking advice on symptom management and medication. One-third of all calls from health professionals were escalated to a palliative care medical consultant. PCAS prevented calls to emergency services  in 10% of cases, and 82% of callers reported their issue was ‘very much’ or ‘completely’ addressed by PCAS. PCAS was shown to be frequently used by the public and healthcare professionals supporting patients with advanced, life-limiting illnesses. The service provided a solution without requiring complex technology, delivering a rapid connection for consumers with specialist palliative care expertise. |
| Mark et al., 2013 | USA | Feasibility study with randomized controlled trial design | Patients with COPD | Skype meetings and videoconferencing technology system | This feasibility study measured the effect of PLB training delivered over Skype on dyspnea, physical activity, health-related quality of life, and self-efficacy. The intervention was found to be feasible and demonstrated marginal improvements in quality-of-life measures. However, when controlling for degree of breathlessness with activity, dyspnea, activity levels, and quality-of-life measures were significantly different, suggesting that as dyspnea worsens, training PLB may be more effective. |
| McCreedy et al., 2022 | USA | Cluster-randomized controlled trial | Nursing home residents with advanced illness | Advance Care Planning (ACP) Video Program | Compared to usual care, an ACP Video Program intervention increased documented Do Not Hospitalize orders among residents but did not significantly reduce hospitalizations. |
| Merz et al., 2022 | USA | Single-site pilot feasibility randomized trial | Patients with advanced cancer | The Digital Supportive Care Awareness and Navigation (D-SCAN) mobile application, a supportive care mobile application intervention | Usability/satisfaction was high for patients and caregivers. Intervention patient and caregiver resource awareness, supportive care resource utilization, patient activation and QOL increased. |
| Middleton-Green et al., 2019 | United Kingdom | Evaluation | Patients thought to be in the last year of life who are not known to specialist palliative care | Gold Line service, a 24/7 single point of contact for any community patients identified to potentially be in their last year of life. It offers care co-ordination, advice and support to the patients and their carers, provided by a nursing team based at the teleconsultation hub. | 4533 telephone calls and 573 video consultations were received from 1813 individuals. 39% of the 5106 calls were resolved by Gold Line team without referral to other services. Interviews with patients and carers reported experiences of support and reassurance and the importance of practical advice. |
| Milbury et al., 2020 | USA | Pilot Randomized Controlled Trial | patients with metastatic non-small cell lung cancer (NSCLC) | couple-based meditation (CBM) intervention via videoconference delivery. A brief intervention that integrates meditation training with emotional sharing and communication exercises | Although attendance was high in both groups, dyads in the CBM group indicated greater benefit of the sessions than those in the control group. Compared with the control group, patients in the CBM group reported significantly lower depressive symptoms and marginally reduced cancer-related stress. Medium effect sizes in favor of the CBM compared with the SE group for depressive symptoms and cancer-related stress were found. Spouses in the CBM group reported significantly lower depressive symptoms. |
| Misplon et al., 2022 | Belgium | Mixed-method evaluation | Lung cancer patients | Digital platform for the collection of patient-reported outcomes and the standardisation of care pathways | Based on the pilot study, we conclude that questions are clear and the platform is user-friendly for 90% of patients in the pilot. The interviews revealed that the weekly follow-up has a positive impact on the patient–provider communica- tion and makes it easier to discuss psychological and palliative care needs. |
| Mooney et al., 2023 | USA | Randomized controlled study | Patients and caregivers in home hospice care for cancer | Automated mHealth, interactive voice response (IVR) system, Symptom Care at Home (SCH), to reduce patient symptom burden. | The intervention produced a mean overall symptom reduction benefit. There was a 38% reduction in days reporting moderate-to-severe patient symptoms compared to usual care with 10/11 symptoms significantly reduced in SCH compared to usual care. |
| Morgan et al., 2017 | Australia | Case report | Patient with advanced non-small cell lung cancer and informal caregiver | Self-reporting of symptoms using telehealth, videoconferencing with palliative care team | Self-reporting was perceived as a means to communicate clinical needs without being a bother to the community palliative care team. Videoconferencing was highly valued as an effective way to communicate and also because it eliminated the need for travel. Patients and carers can engage in telehealth supported palliative care and the empowerment enabled by this additional form of communication with the health service. |
| Moscato et al., 2021 | Italy | Pre-post single-arm study | Advanced cancer patients | VR headset used in psychophysical discomfort | Immediate effect on anxiety, depression and pain levels after each session. No significant change pre-post intervention. The VR intervention was appreciated by participants, and no one reported adverse side effects caused by its use. |
| Nemecek et al., 2019 | Austria | Feasibility study | patients with advanced cancer and their family caregiver | telemedical device enabling patients encountering medical problems to send a direct request to a palliative care team | This study showed a good feasibility despite the low overall willingness to participate in a relatively “technical” trial. The hospital anxiety and depression scale (HADS) was significantly lower in the intervention group, suggesting an improved quality of life. Although a decrease in the number of hospital admissions could not be shown, the user satisfaction was very good. |
| Ngoma et al., 2021 | Tanzania | Randomized controlled study?? | Adult patients with incurable cancer | mPalliative Care Link (mPCL), remote symptom-focused assessment and response-based care coordination | Measures of physical and emotional symptoms were higher (reflecting higher symptom burden) in the mPCL arm, likely reflecting the lack of a true usual-care arm and between-group clinical and sociodemographic differences; however, patient satisfaction with the care provided was high in both arms. This work holds promise for closing a large palliative care gap among patients with cancer in under resourced settings globally. |
| Oelschlagel et al., 2023 | Norway | Qualitative study with a longitudinal, exploratory design | Cancer patients living at home | The application Remote home care (RHC), a service that enables health care professionals to remotely monitor and manage patients' safety, security, wellness, treatment and care | Three themes were identified: (1) potential to facilitate self-governance of life-limiting illness in daily life, (2) need for interpersonal relationships and connections, and (3) experiences of increased responsibility and unclear utility of the Remote Home Care. |
| Olesen et al.,2022 | Denmark | Qualitative cross-sectional design | Family caregivers of people with ALS and cognitive and/or behavioral impairments | EMBRACE, an intervention with blended learning format, combining both videos and virtual group meetings. | Results indicate that the intervention supported caregivers in dealing with everyday challenges and reduced their experience of guilty conscience, fear, loneliness, uncertainties, and gave insights into ways of dealing with everyday challenges now and  in the future that they could not have gained elsewhere. A special atmosphere in the group meetings fostered greater social connectedness and feelings of belonging to  a group among the participants, thereby reducing feelings of loneliness. |
| Palma et al., 2021 | Chile | Pilot study | hospitalized patients referred to a mobile palliative care team and their relatives | telehealth system with synchronic videoconferences | Implementation of a spiritual and psychological palliative intervention via telehealth produced a high percentage of acceptance. The main intervention was psychological support to relatives of severely ill patients with withdrawal or with-holding of life-sustaining treatment. The quality of audio and video images was rated as good or very good. Of the users, 100% indicated they would use the intervention again, 98% would recommend it to others. Limitations identified in 5 % of sessions were interruption due to episodic loss of internet signal, lack of availability of internet connection, audio or video difficulties. |
| Parker et al., 2021 | Australia | Evaluation with surveys | Family carers looking after someone with a life limiting illness | Carer-Help online toolkit for family carers looking after someone with a life limiting illness. provides essential information on how to provide care to a partner, family member, or friend at the end-of-life. | The results indicate that CarerHelp is a useful resource, from the perspective of health professionals. Although only a proxy rating for family carers, health professionals all rated CarerHelp as useful in increasing family carer preparation for the caring role, including undertaking physical tasks and providing emo- tional support. In addition, they rated that CarerHelp would increase family carers’ knowledge of services, their confidence to care and ability for self-care. |
| Pasanen et al., 2022 | Australia | Qualitative interview study | Palliative care patients and physicians | Telehealth consultations | Telehealth transformed the ways physicians and patients in this study perceived and engaged with outpatient palliative care across the entire continuum of care. Four key themes were identified: (1) broadened access to palliative care; (2) delivery of care, facilitating continuity of care; (3) engagement with care; and (4) the future, proposed a hybrid mix of telehealth and in-person appointments moving forward. |
| Paul et al., 2019 | Canada | Descriptive, exploratory, proof-of-concept study | Older rural patients with life-limiting illness | Mobile web-based videoconferencing | Using WBVC reduced the burden and expense of travel for patients, families, and consultants, and increased consultants’ efficiency and productivity. Analysis of qualitative data revealed four themes: communication, logistics, technical issues, and trust. Participants reported they were comfortable discussing concerns by WBVC and felt it was an acceptable and convenient way to address needs. Audiovisual quality was not ideal but was adequate for communication. Use of WBVC improved access and saved time and travel. Fears were expressed about lack of security of information transmitted over the Internet |
| Potter et al., 2015 | Canada | Evaluation | Nurses and unlicensed care providers | An educational intervention designed to improve rural nurses and unlicensed care providers’ confidence in a palliative approach to care. | Participants valued the joint interactive education and came away with greater appreciation for one another’s’ contributions to care. Insights were gained into common challenges when attempting to apply a palliative approach in rural areas. Important lessons were learned about educating nurses and unlicensed care providers together, about the use of technology for this group, and about teaching the concept of a palliative approach. Educating through distance technology in the rural context was problematic. Technology problems and the demands of home life had a distracting effect on participants. Learners preferred face-to-face encounters, particularly with the emotionally laden content of palliative care. Although distance technology is commonly seen as the solution to educating in rural and remote areas, innovative solutions that connect learners to one another in their home communities and work sites may be more acceptable to rural nurses. If technology is used, the adage “keep it simple” will provide the best outcomes. |
| Qiaohong et al., 2017 | Canada | Feasibility study with a cross-sectional survey design | Palliative in-patients at end of life and their family members | Keep in Touch (KIT). Internet-based communication and information technologies at the bedside in in-patient palliative care for communication with family and friends. | Palliative patients and family members used the technology to keep in touch with family and friends, entertain themselves, look up information, or accomplish tasks. Most participants found the technology easy to use and reported that it helped them feel better overall, connected to others and calm. The availability of competent, respectful, and caring technical support personnel was highly valued by patients and families. Health care providers identified that computer technology helped patients and families keep others informed about the patient’s condition, enabled sharing of important decisions and facilitated access to the outside world. |
| Rafter, 2016 | United Kingdom | Case study | End-of-life patients with pressure ulcers | E-health system to provide a care pathway for end-of-life patients with risk of pressure ulcers. | The care pathway from the e-health system allowed the hospice staff to deliver effective care for patients’ pressure ulcers. There was some improvement in pressure ulcers despite the patients being near the end of their lives. All patients received their care pathways within 24 hours of referral on  the e-health system. Twenty members of staff gave feedback that was very positive, noting they found the system very easy to use as it employed a standardized approach. They also commented that it enhanced their job satisfaction, as it resulted in effective care  and good patient experiences. |
| Rainsford et al., 2022 | Australia | Hybrid type-3 effectiveness-implementation study | Staff in rural residential aged care | Needs rounds via telehealth | While face-to-face meetings were preferred, videoconferencing was readily implemented and an acceptable compromise; telephone was a suitable backup. Telehealth proved to be an effective platform to continue Needs Rounds in existing sites and initiate them in new ones. Telehealth Needs Rounds were useful in facilitating end-of-life care planning. Telehealth delivery was as effective as face-to-face modes, in terms of numbers of attendees and residents discussed, and completion of end-of-life care plans. |
| Rochmawati et al., 2022 | Indonesia | Feasibility study with intervention and control group | Family caregivers in palliative care | The mobile app, My Semah, developed to address family caregivers’ needs. My Semah stands for management of symptoms at home. | Mobile app health education improved the caregiver readiness for the intervention group. Our findings demonstrate feasibility and acceptability for delivering education to family caregivers through a smartphone app |
| Rosa et al., 2022 | USA | Qualitative interview study | Palliative care specialists and cancer patients | Telehealth services | Several themes were identified, including the barriers related to telehealth, the impact of telehealth on the quality of relationships with patients, their families, and coworkers, and the changes in perceived self-efficacy of fulfilling job responsibilities. Participants reported both positive and negative inpatient telepalliative care experiences associated with various domains of professional functioning, such as communication, relationships with key stakeholders, and self-efficacy. |
| Royackers et al., 2016 | Canada | Pilot study | Adults requiring in-home palliative care in end-of-life care, and family caregivers | The eShift model of home care delivery with point-of-care technology along with remotely located registered nurses and personal support workers in the client's home. | Four themes were identified: the health care family; making the invisible visible; there’s no place like home; and burden of love. Overall, informal caregivers indicated that they were very satisfied with care delivery, felt supported by health care providers, and were able to support their family member to die at home. |
| Samara et al., 2021 | Australia | Quality improvement | Care homes | Telehealth via readily available platforms of Zoom, WebEx, FaceTime, and Skype | There was no statistical difference between face-to-face and telehealth groups in the average number of care home residents being discussed at Needs Rounds, anticipatory medication pre-scribing, GP participation, and completion of ACP at case conferencing. This implies that telehealth Needs Rounds and case conferences are as effective as face-to-face for identifying residents at risk of dying and planning for end-of-life care. |
| Schuler et al., 2023 | Australia | Feasibility mixed methods study | patients and family caregivers | A wearable sensor (WS)-triggered ecological momentary assessments (EMAs) and electronic patient-reported outcome. | Daytime sensor wear-time had 73% adherence. Participants perceived value in this support. Quantity and severity of ‘‘stress’’ events were higher in patients. Sleep disturbance was similar but for different reasons: patients (physical symptoms) and caregivers (worrying about the patient). |
| Schuit et al., 2022 | Netherlands | Randomized controlled trial | Family caregivers of incurable ill cancer patients | The eHealth self-management application Oncokompas to support partners of incurably ill cancer patients to adopt an active role in improving their own HRQOL and to find optimal supportive care if needed | The reach, in terms of eligibility and participation rate, was estimated at 83–91%. Partners were most likely reached via palliative care consultants, patient organizations, and palliative care networks. In the one-and-a-half-year recruitment period and via the 101 organizations involved, 58 partners were included. There were no significant effects of Oncokompas on caregiver burden, self-efficacy, or HRQOL. |
| Shirley et al., 2021 | USA | Pilot study | Veterans receiving palliative care services | Telemedicine, VA Video Connect (VVC) application | The pharmacotherapy implementation rate was 60% including a deprescribing rate of 57%, decreasing polypharmacy and potentially resulting in a decrease in adverse drug events. Veterans were on average very satisfied with their visit. |
| Soliman et al., 2023 | USA | Retrospective electronic record review | Healthcare professionals | Tele-health delivered palliative care | PC consultations were more likely for goals of care or hospice during peak-COVID compared to pre-COVID. Rates of assessment of physical and psychological symptoms were lower during peak relative to pre-COVID and post-peak periods. There were no differences in assessment of patients’ social needs, family burden, or goals of care across periods. |
| Takahashi et al., 2012 | USA | Randomized controlled trial | Older adults with chronic health problems and considered at high risk for future hospital admission | Telemonitoring with the Intel® Health Guide | More patients enrolled in hospice care overall, with 9 of 94 participants (9.6%) in the telemonitoring group enrolling in a hospice, compared to 4 of 100 subjects (4.0%) enrolling in a hospice in the usual care group (P = 0.12). There were no significant differences found between the groups regarding time until entry into hospice. |
| Tasneem et al., 2019 | USA | Qualitative interview study | Patients in palliative care | Telemedicine video visits | Despite concerns over truncated physical exams and prescription limits, the majority of patients favored having the opportunity for telemedicine video visits, felt that the doctor-patient relationship would not suffer, had confidence in their or their surrogate’s technical abilities to navigate the video visit, had privacy concerns on par with other technologies, had few cost concerns, and believed a video alternative to an in-person visit might increase access, save time as well as increase comfort and safety by avoiding a trip to the office. |
| Taubert et al., 2018 | United Kingdom | Mixed-method design service review | People with palliative and life-limiting illness | Videos, Talk CPR, to improve communication in do not attempt cardiopulmonary resuscitation decisions in palliative illness | Videos were demonstrably acceptable to both patient and carer groups, and improved healthcare professional confidence and understanding. Videos went live on the TalkCPR website, in all Welsh Health Boards and on Youtube, and are now used in routine practice throughout Wales. |
| Teter et al., 2021 | USA | Quasi-experimental design | Patients in palliative care | Accessibility to a care provider via telemedicine application | A reduction in the number of preintervention and postintervention ED visits and 911 calls was noted among participants in the intervention group, from 12 (54.5%) to one (4.5%). A telemedicine hospice care application may benefit a palliative and hospice organization by enhancing patient clinical outcomes and decreasing emergency department visit rates. |
| Tumeh et al., 2023 | Brazil, USA | a quasi‐experimental pre‐post study | Patients with advanced cancer | The Comfort platform was developed to assess emotional and physical symptoms among patients. | patients who engaged with the program reported improvement in physical and emotional symptoms, as well as in their overall quality of life, compared with patients who did not engage with the program. |
| van Gurp et al., 2015 | Netherlands | Qualiative study | Home-based palliative care patients | Weekly real-time audiovisual teleconsultations | Three key concepts described the impact of teleconsultation on the patient-professional relationship in palliative homecare: transcending the institutional walls of home and hospital; transparency of teleconsultation technology; and technologized, intimate patient-professional relationships. Teleconsultation offers (1) condensed encounters between home-based palliative care patients and distant professionals, (2) a unique insight into the patients’ daily lives for palliative care specialists, and (3) long-term interaction that results in trustful relationships and experiences of intimacy and relief. |
| van Gurp et al., 2016 | Netherlands | Qualitative study | Home-based palliative care patients, hospital-based specialist palliative care team clinicians, and primary care physicians | Teleconsultations | Analysis showed that the introduction of specialist palliative care team-patient teleconsultation led to collaboration between  primary care physicians and specialist palliative care team clinicians. In most cases, interprofessional contact was restricted to backstage work after teleconsultation. In one deviant case, both the patient and the professionals were simultaneously connected through teleconsultation. Two themes characterized integrated palliative care at home as a consequence of teleconsultation:  (1) professionals defining responsibility and (2) building interprofessional rapport. |
| Viitala et al., 2021 | Finland | Qualitative interview study | Adult patients suffering from incurable cancer | Mobile application for symptom monitoring and support coping with disease | The majority of the interviewees viewed the mobile application as helpful for monitoring relevant symptoms and coping with the disease. The patients’ sense of security was increased by their ability to contact the clinic at all times. As a communication channel, the mobile application was seen as more convenient than the telephone, and it provided a sense of freedom for the patients as contacting the clinic was not tied to time or place. The patients also experienced increased involvement with their own care and had a sense of staying abreast with their treatment. A minority of the interviewees reported that there was a certain disease-centredness  in using the mobile application. |
| Watanabe et al., 2013 | Canada | Pilot study | Patients in palliative care in rural areas | Telemedicine, telecommunication video consultations | Forty-four initial consultation and 28 follow-up visits took place. Anxiety and appetite were statistically significantly improved at the first follow-up visit . Average per visit savings for patients seen by telehealth versus attending the CCI were 471.13 km, 7.96 hours, and Cdn $192.71, respectively. Patients and referring physicians indicated a high degree of satisfaction with the clinic. |
| White et al., 2019 | United Kingdom | Prospective longitudinal cohort study | Community hospice nurses | Teleconferencing technology to support and train healthcare providers remotely | Mean knowledge score improved significantly as did overall self-efficacy scores. There was no significant difference in self-efficacy. 96% recorded gains in learning, and 90% felt that the project had improved the care they provided for patients. 83% would recommend the project to other HCPs. 70% stated the technology used in the project had given them access to education that would have been hard to access due to geography. |
| Wilkie et al., 2020 | USA | Randomized conrolled study | Patients with cancer in hospice care | Internet-based pain management application to improve cancer pain outcomes (AINRelieveIt). | The effect on analgesic adherence was not significant. Post-test worst pain intensity was significantly higher for the experimental group, but the difference was not clinically meaningful. There was nearly universal availability of prescriptions for strong opioids and adjuvant analgesics for neuropathic pain in both groups. Lay caregivers’ pain misconceptions were significantly lower in the experimental group than the usual care group. |
| Zeiser, 2023 | USA | Evidence-based quality improvement (QI) | Veterans in palliative care | Music therapy telehealth services | Results from the QI survey indicated that music therapy telehealth provided affective and interpersonal benefits, and increased compliance with other therapies. Reflections from key stakeholders included clinical recommendations for service implementation and descriptions of the influence of the telehealth modality on the therapeutic relationship. Findings suggested that music therapy telehealth services can provide psychosocial support for veterans, families, and staff in palliative care. |
